# Elevated S100A9 expression in tumor stroma functions as an early recurrence marker for early-stage oral cancer patients through increased tumor cell invasion, angiogenesis, macrophage recruitment and interleukin-6 production

**DOI:** 10.18632/oncotarget.4951

**Published:** 2015-07-22

**Authors:** Wei-Yu Fang, Yi-Wen Chen, Jenn-Ren Hsiao, Chiang-Shin Liu, Yi-Zih Kuo, Yi-Ching Wang, Kung-Chao Chang, Sen-Tien Tsai, Mei-Zhu Chang, Siao-Han Lin, Li-Wha Wu

**Affiliations:** ^1^ Institute of Basic Medical Sciences, College of Medicine, National Cheng Kung University, Tainan, Taiwan, R.O.C; ^2^ Institute of Molecular Medicine, College of Medicine, National Cheng Kung University, Tainan, Taiwan, R.O.C; ^3^ Department of Pharmacology, College of Medicine, National Cheng Kung University, Tainan, Taiwan, R.O.C; ^4^ Department of Otolaryngology, National Cheng Kung University Hospital, Tainan, Taiwan, R.O.C; ^5^ Department of Pathology, National Cheng Kung University Hospital, Tainan, Taiwan, R.O.C; ^6^ Department of Radiation Oncology, National Cheng Kung University Hospital, Tainan, Taiwan, R.O.C

**Keywords:** S100A9, oral cancer, recurrence, IL-6, macrophages

## Abstract

S100A9 is a calcium-binding protein with two EF-hands and frequently deregulated in several cancer types, however, with no clear role in oral cancer. In this report, the expression of S100A9 in cancer and adjacent tissues from 79 early-stage oral cancer patients was detected by immunohistochemical staining. Although S100A9 protein was present in both tumor and stromal cells, only the early-stage oral cancer patients with high stromal expression had reduced recurrence-free survival. High stromal S100A9 expression was also significantly associated with non-well differentiation and recurrence. In addition to increasing cell migration and invasion, ectopic S100A9 expression in tumor cells promoted xenograft tumorigenesis as well as the dominant expression of myeloid cell markers and pro-inflammatory IL-6. The expression of S100A9 in one stromal component, monocytes, stimulated the aggressiveness of co-cultured oral cancer cells. We also detected the elevation of serum S100A9 levels in early-stage oral cancer patients of a separate cohort of 73 oral cancer patients. The release of S100A9 protein into extracellular milieu enhanced tumor cell invasion, transendothelial monocyte migration and angiogenic activity. S100A9-mediated release of IL-6 requires the crosstalk of tumor cells with monocytes through the activation of NF-κB and STAT-3. Early-stage oral cancer patients with both high S100A9 expression and high CD68+ immune infiltrates in stroma had shortest recurrence-free survival, suggesting the use of both S100A9 and CD68 as poor prognostic markers for oral cancer. Together, both intracellular and extracellular S100A9 exerts a tumor-promoting action through the activation of oral cancer cells and their associated stroma in oral carcinogenesis.

## INTRODUCTION

Oral cancer is the eighth most common cancer worldwide [1]. Squamous cell carcinoma (SCC) constitutes 90% of all oral malignancies [2]. There are approximately 275,000 new cases and over 120,000 deaths associated with oral SCC every year [3]. The survival rate for the advanced disease drops drastically from > 75% to < 20% for those in the earliest stage [4]. The lack of improvement in 5-yr survival rate in the last 3 decades indicates that tumor size, lymph node involvement and clinical stage, which are considered as the markers for disease aggressiveness, do not sufficiently account for the variability in clinical outcomes for such patients [3]. Moreover, oral cancer patients with similar clinical manifestations often have distinct clinical outcomes. Identifying an early prognostic marker for oral cancer will not only complement the existing paradigms in assessing disease aggressiveness and prognosis, but also help the doctors fine-tune the existing therapeutic regimens, consequently leading to improving patient clinical outcome.

S100A9 is a calcium- and zinc-binding molecule of S100 family, and expressed in cells of myeloid origin [5]. Upon ion binding, S100A9 undergoes conformational change and becomes an efficient ligand for pro-inflammatory receptors including receptor for advanced glycation end products and Toll-like receptor 4 (TLR4) [6]. S100A9 homo- or hetero-dimerizes with S100A8, another S100 family member [7]. The expression of S100A8 but not S100A9 is essential for mouse survival [8, 9]. Moreover, the absence of S100A9 or TLR4 expression delays tumor incidence in a spontaneous prostate cancer model [10]. S100A9 but not S100A8 overexpression promoted tumor growth [11]. S100A9 also binds to heparin sulfate, mediates neutrophil adhesion to fibronectin, and increases beta 2 integrin Mac-1 affinity on neutrophils [12, 13]. These data together support a distinct role of S100A9 from S100A8 in animal development and tumorigenesis.

In addition to its up-regulation in several epithelial tumors, S100A9 is also abundantly expressed in tumor infiltrating immune cells [14]. Elevated serum levels of S100A9 protein are found in the patients suffering from inflammatory diseases like rheumatoid arthritis and colitis, and the increase is associated with the disease severity [15, 16]. Tumor microenvironment exerts a key influence on tumor progression and metastasis, and re-shaping its characteristics may offer unexpected therapeutic benefits for cancer treatment. The mediators and cellular effectors of inflammation are thus important constituents of tumor microenvironment [17, 18]. Although a number of putative functions have been proposed for S100A9, its biological role, particularly, in oral cancer cells remains elusive. The purpose of this study is to investigate the role of S100A9 deregulation in oral cancer cells and their stroma.

## RESULTS

### Association of increased stromal S100A9 expression with poor clinical outcome of early-stage oral cancer patients

**Figure 1:**
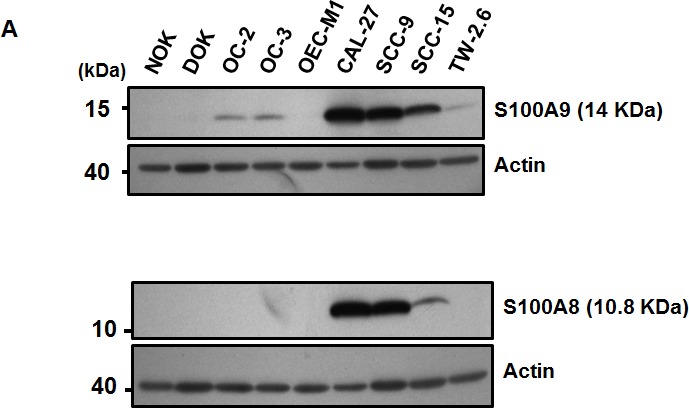

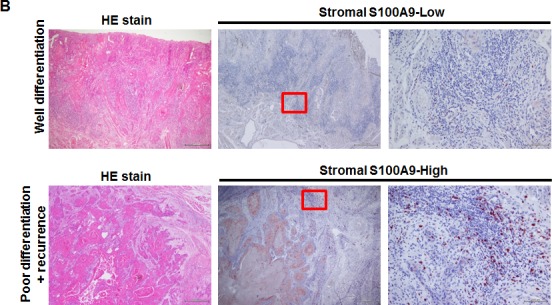

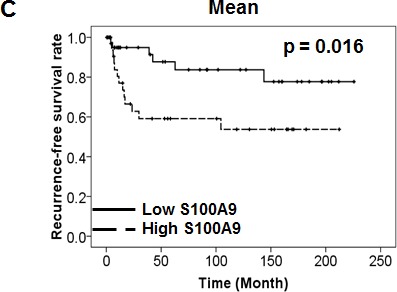
Frequent alteration of S100A9 protein in oral cancer and its impacts on patient clinical outcome **A.** The expression of S100A9 and S100A8 protein in NOK, DOK and 7 oral cancer cell lines, respectively, detected by Western Blot analysis. Actin, a loading control. **B.** The staining of S100A9 protein in the tumor and stroma of two representative oral cancer specimens, respectively, with high and low stromal expression by IHC staining. Left panels, HE staining; Middle and Right panels, IHC staining of S100A9 and the enlargement of red box on the Middle panel (40X) highlighting the border between tumor and stroma in Right panel (200X). **C.** Kaplan-Meier analysis of recurrence-free survival for high and low stromal S100A9 groups. All the 79 patients were divided into two groups based on the mean expression of S100A9 in the tumor stroma. High, greater than mean. Low, equal to or less than mean.

To examine if S100A9 protein was deregulated in a panel of oral cancer lines, Western blot analysis was used. The expression of S100A9 protein was differentially increased in 6 out of 7 oral cancer lines relative to NOK and dysplastic oral keratinocytes (DOK) (Figure 1A, Top). Although S100A9 could form a complex with S100A8, S100A8 protein was mainly detected in CAL-27, SCC-9 and SCC-15 cells (Figure 1A, Bottom). We confirmed no cross-reactivity between S100A8 and S1009 antibodies used in this study (data not shown). Immunohistochemical (IHC) staining was used to study the relation of S100A9 expression with the clinicopathologic characteristics and clinical outcome of 79 early-stage oral cancer patients. S100A9 was detected in both tumor cells and stroma (Figure 1B). We divided these patients into 2 groups, high and low, based on the mean S100A9 staining intensity in tumor or stroma. High S100A9 expression in stroma but not in tumor cells was significantly associated with non-well differentiation and recurrence (Table 1). Although stromal S100A9 deregulation had no impact on patient overall survival (Figure S1), high stromal S100A9 patients had significantly shorter recurrence-free survival than those with low expression (Figure 1C, *p* = 0.016). By contrast, no significant impact of stromal S100A8 deregulation on patient recurrence-free survival was detected (Figure S2). Together, S100A9 deregulation in tumor stroma may serve as an early poor prognosis marker and have a role in tumor recurrence.

**Table 1:** Mean stromal S100A9 expression in relation to clinicopathologic characteristics of early-stage oral cancer

		S100A9 expression^a^		
		Low	High	
	Total (*n* = 79)	*n* = 45 (57.0%)	*n* = 34 (43.0%)	*P* value
Median age (yr)				
<48	40	21 (52.5)	19 (47.5)	0.417
<48	39	24 (61.5)	15 (38.5)	
Tumor site				
Buccal + Tongue	63	38 (60.3)	25 (39.7)	0.232
Others	16	7 (43.8)	9 (56.3)	
Differentiation				
Well	52	34 (65.4)	18 (34.6)	0.036*
Moderate + Poor	27	11 (40.7)	16 (59.3)	
Recurrence				
No	60	39 (65.0)	21 (35.0)	0.010*
Yes	19	6 (31.6)	13 (68.4)	
2nd primary				
No	73	40 (54.8)	33 (45.2)	0.175
Yes	6	5 (83.3)	1 (16.7)	

* *P* < 0.05 by Chi-square test

a The mean staining intensity quantified by HistoQuest was 15.14 arbitrary units. High, greater than or equal to mean. Low, less than mean.

### Ectopic S100A9 expression primarily stimulated oral cancer migration and invasion

**Figure 2:**
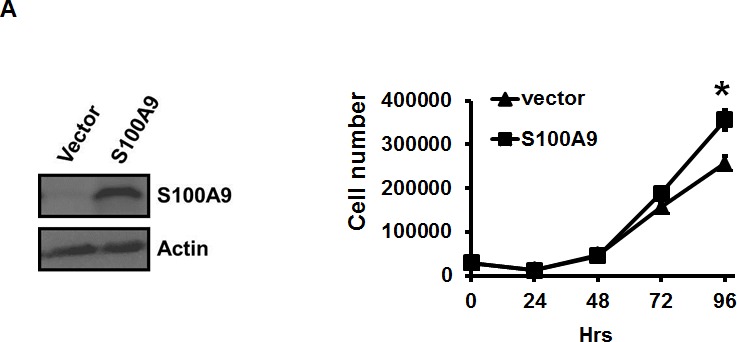

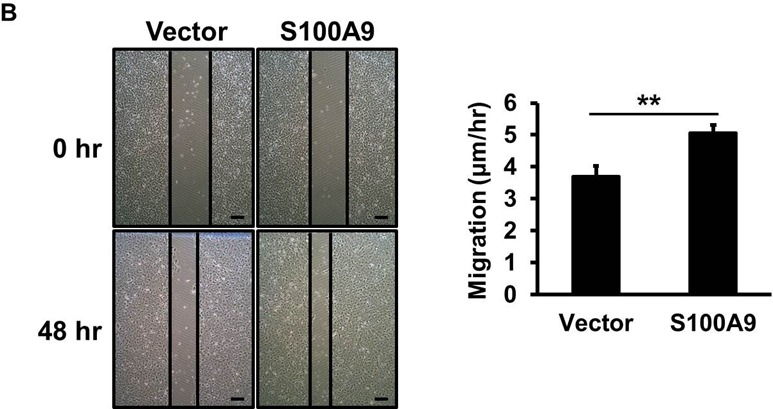

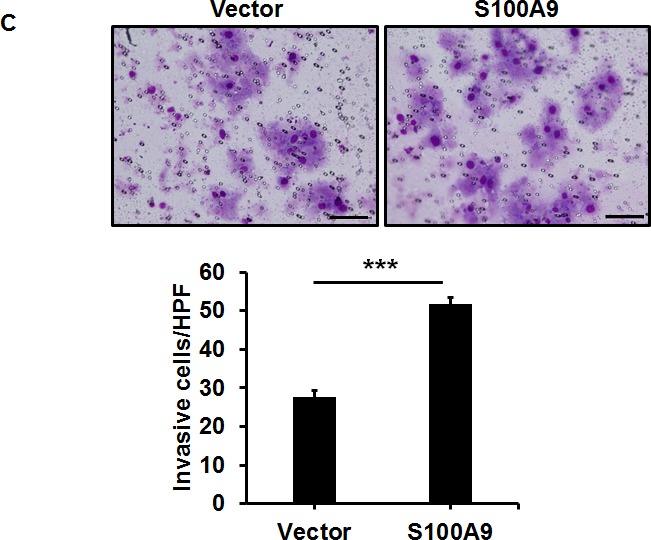

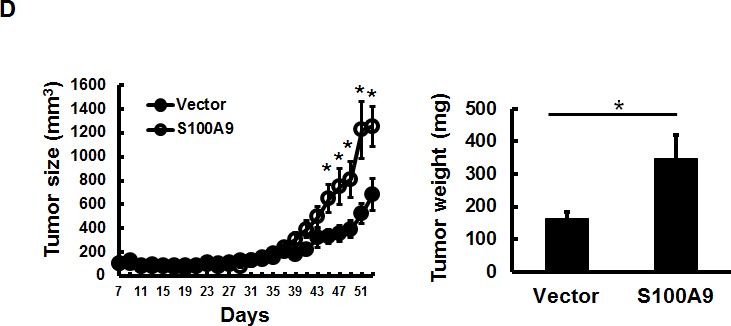

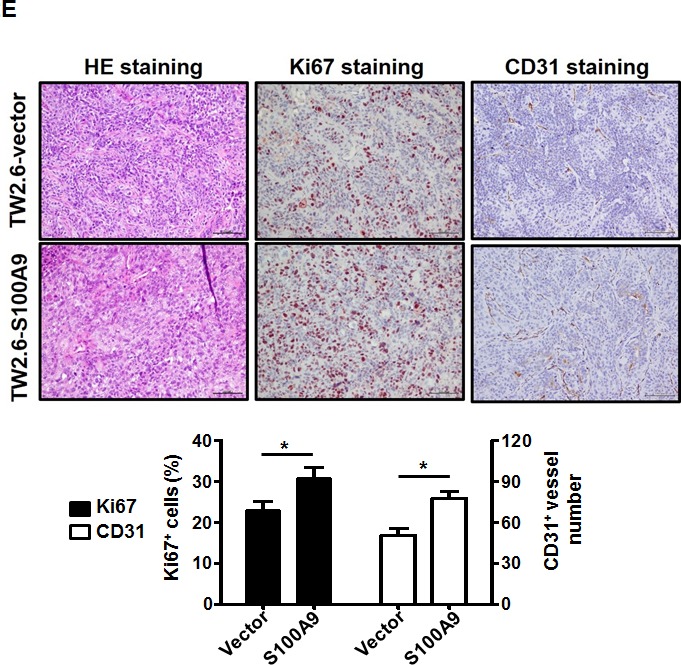

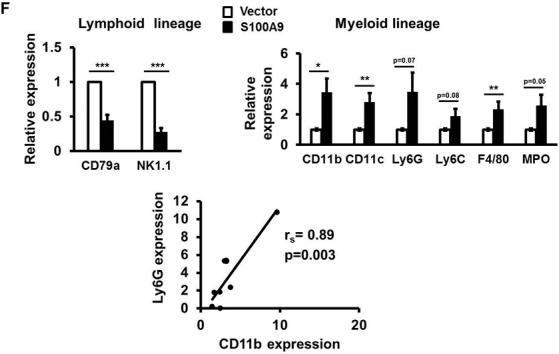

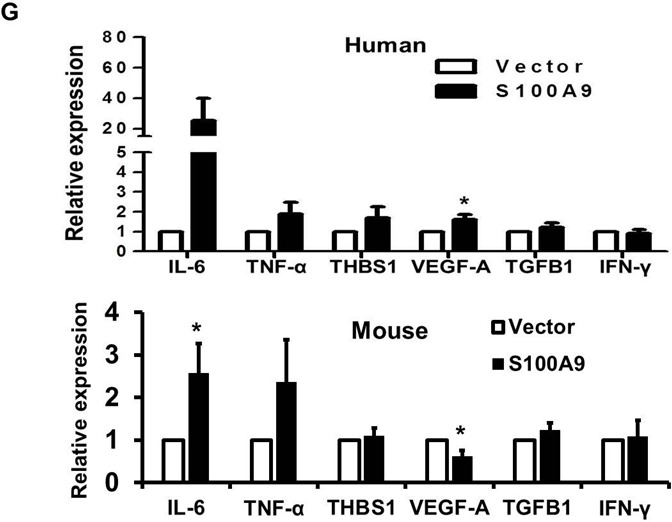
Pro-tumorigenic effect of tumor-derived S100A9 *in vitro* and *in vivo* **A.** TW-2.6 cells were infected with lentiviruses expressing human S100A9 or empty vector as a negative control. Left, S100A9 expression was measured by Western Blot analysis and actin was a loading control. Right, viable cell numbers were enumerated by cell proliferation assay. **B.**, **C.** Cell migration and invasion abilities were, respectively, measured by wound healing and cell invasion assays. Data are the mean ± SEM. The representative images for each assay were also shown. Scale bar, 100 m. **D.** Left, S100A9- or vector- expressing TW-2.6 cells were subcutaneously injected into male nude mice (8 mice for each group). Tumor sizes were measured every 2 days for 53 days due to low tumorigenic potential of TW-2.6. Right, mean tumor weights of vector or S100A9 group at the end point (*N* = 8). **E.** Top, Representative HE and IHC staining of Ki67 and CD31 in TW-2.6-vector or -S100A9 tumors (200X magnification). Five random fields (200X) of the Ki67+ nuclei or CD31+ microvessels for each mouse tissue were counted and averaged. Data are Mean±SEM. **F.** The expression of tumor infiltrating immune cell markers in each tumor tissue was analyzed in triplicate by qRT-PCR. Lymphoid lineage markers: CD79a and NK1.1. Myeloid lineage markers: CD11b, CD11c, Ly6G, Ly6C, F4/80 and MPO. Pearson correlation analysis showing a significant association of CD11b with Ly6G expression in S100A9-bearing xenografts (Bottom). **G.** Differential expression of the chemokines and cytokines in each xenograft tumor was analyzed in triplicate by qRT-PCR. Top, human-specific probes. Bottom, mouse-specific probes. All qRT-PCR data are mean±SEM (8 mice per group). *p < 0.05, **p < 0.01, ***p < 0.001 *versus* vector control.

Since S100A9 was detected in tumor cells, we ectopically expressed S100A9 in two low- S100A9 oral cancer lines, TW-2.6 and highly metastatic HSC-3, with distinct tumorigenic potential in nude mice (Figures S3-4). Western blot analysis confirmed the increase of S100A9 protein in the stable clones (Figures 2A and S5A, Left panels). Ectopic S100A9 increased TW-2.6 cell proliferation, migration and invasion (Figure 2A–2C). The promoting effect on cell migration and invasion but not proliferation was also detected in HSC-3 line ectopically expressing S100A9 (Figure S5A-B). The stimulatory action of tumor S100A9 was mainly on cell migration and invasion.

### Tumor S100A9 promoted tumorigenesis *in vivo* accompanied with the differential expression of immune cell markers and cytokines

To examine the effect of ectopic S100A9 expression on xenograft tumorigenesis, we subcutaneously injected S100A9-expressing or vector control TW-2.6 cells onto male nude mice (8 mice per group). Ectopic S100A9 promoted TW-2.6 tumor size with time (Figure 2D, Left). The mean tumor weight, the percentage of proliferating Ki67-positive nuclei, and CD31-positive microvessel numbers were significantly increased in S100A9 tumor tissues relative to vector ones at the ending point (Figure 2D–2E). Consistent with no stimulation of high tumorigenic HSC-3 proliferation *in vitro*, S100A9 had no tumor promoting effect on these tumors (Figures S5C). S100A9 can function as one of damage-associated molecular patterns involved in regulating immune response and inflammation, with the ability to induce IL-6 and TNF-α [19]. These two multi-functional cytokines are involved in regulating the immune response, hematopoiesis, and inflammation [20]. We analyzed the expression of lymphoid and myeloid lineage markers, and six cytokines (IL-6, TNF-α, THBS1, VEGF-A, TGFB1 and IFN-γ) in S100A9-bearing tumors by using qRT-PCR. Two lymphoid markers were CD79a (B cells) and NK1.1 (natural killer cells), and 6 myeloid markers were CD11b (monocytes), CD11c (dentric cells), Ly6G, Ly6C, F4/80 (macrophages) and MPO (neutrophils) as reported [21]. Although there was no thymus in nude mice, S100A9 tumors still manifested a significant increase of myeloid lineage markers but a significant reduction of CD79a and NK1.1 expression (Figure 2F). A positive association of CD11b and Ly6G was detected in S100A9-bearing tumors (Figure 2F, r_s_ = 0.89, *p* = 0.003). Among the tested cytokines, there was a strong induction of IL-6 paracrine (mouse probe) and autocrine release (human probe) but not that of the other ones in the S100A9 xenografts (Figure 2G). Together, S100A9 promoted TW-2.6 tumor formation as well as dominant increase of myeloid cell marker and IL-6 expression *in vivo*.

### The expression of S100A9 in monocytes enhanced the migration and invasion of co-cultured oral cancer cells

**Figure 3:**
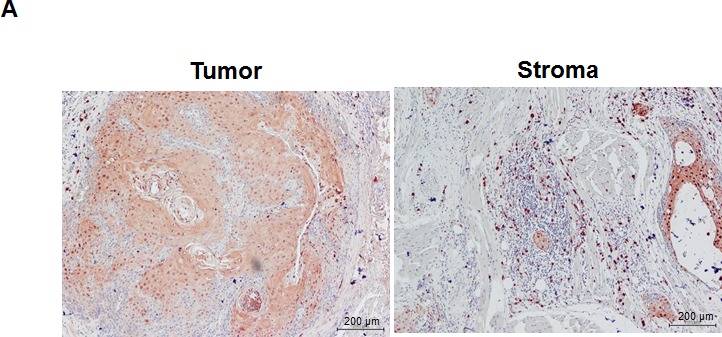

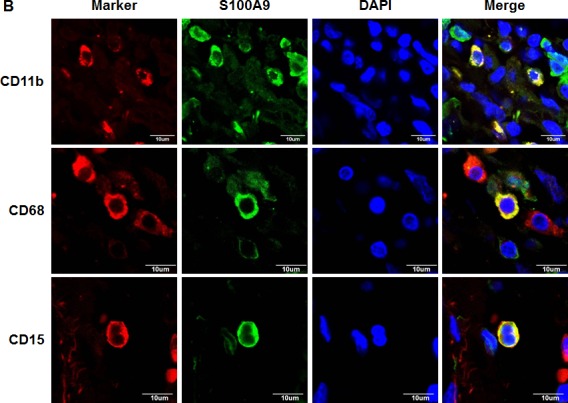

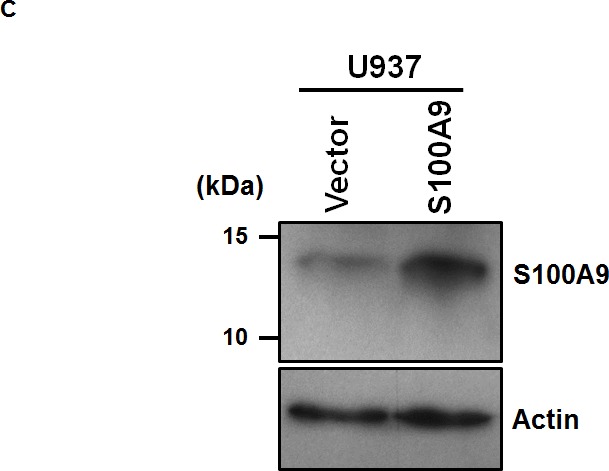

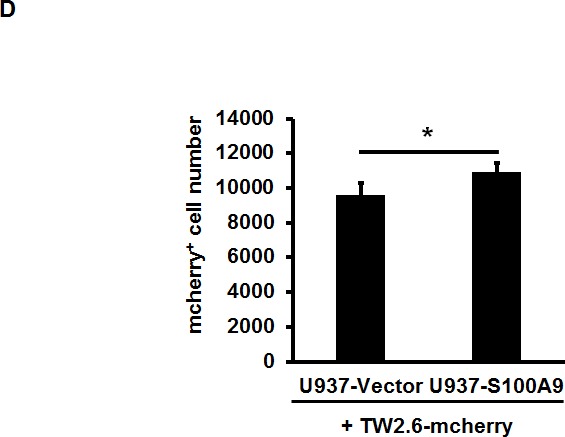

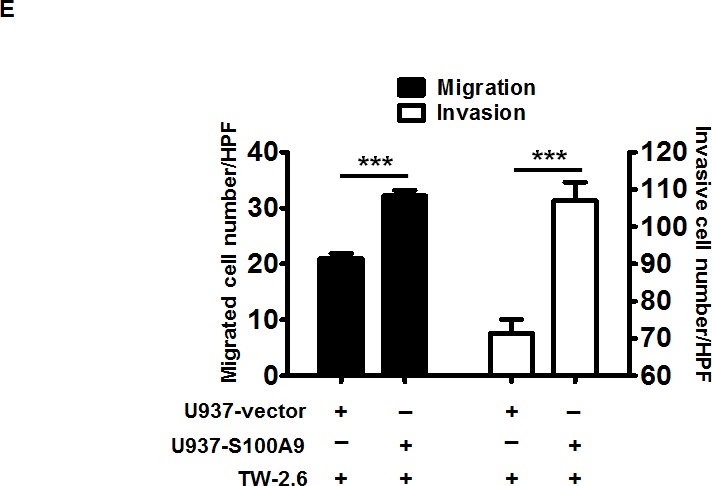
Stromal S100A9 expression in monocytes promotes the migration and invasion of co-cultured oral cancer cells **A.** S100A9 protein was detected both in tumor and stroma of one represent oral cancer specimen by IHC staining (100X magnification). **B.** The presence of S100A9 protein and indicative myeloid cell markers in tumor stroma by confocal immunofluorescence staining of serial sections of the oral cancer specimen. CD15, neutrophils; CD11b, monocytes; CD68, macrophages; DAPI, nuclear stain. **C.** The increase of ectopic S100A9 protein in the indicated U937 cell clones detected by Western blot analysis. Actin serves as a loading control. **D.** TW2.6-mCherry cells were incubated with the indicated U937 cells in 2:1 ratio for 48 hours. The number of mCherry-positive cancer cells was measured by a SpectraMAX M3 microplate reader. Data are mean ± SD. **E.** Cell migration and invasion abilities of TW-2.6 cells co-cultured with indicated U937 cells were measured by using Transwell plates. TW-2.6 cells seeded in the inserts were incubated with the indicated U937 cells in the bottom wells for 24 hours. Data are mean ± SEM. *p < 0.05 or ***p < 0.001 *versus* vector.

In addition to the detection of S100A9 protein in tumor cells, S100A9 was also abundantly expressed in the tumor stroma (Figure 3A). Since S100A9 was originally identified in myeloid cells [22, 23], confocal immunofluorescence microscopy was used to study the type(s) of myeloid cells expressing S100A9 in tumor stroma of clinical specimens. Stromal S100A9 was present in the cells expressing CD11b (monocytes), CD15 (neutrophils), or CD68 (macrophages), and also those without these markers (Figure 3B). Early-stage oral cancer patients with high stromal S100A9 tended to have recurrence relative to the low group (Table 1, *p* = 0.01), suggesting a role of stromal S100A9 in cancer recurrence. We ectopically increased S100A9 expression in monocytic U937 cells as detected by Western blot analysis (Figure 3C). To address if monocytic S100A9 could impact neighboring cancer cell behaviors, we co-cultured mCherry-expressing oral cancer lines with vector- or S100A9-U937 cells for the indicated time followed by the measurement of mCherry-positive oral cancer cell proliferation, migration and invasion. Consistent with the pro-tumor role of S100A9 expression in oral cancer cells (Figures 2 and S5), stromal expression of S100A9 also significantly enhanced oral cancer migration and invasion while differentially regulating oral cancer proliferation in the co-culture experiment (Figures 3D–3E and S6). Together, the expression of S100A9 in monocytes exerts a pro-tumor effect upon co-culturing with oral cancer cells.

### Serum S100A9 levels were differentially elevated in oral cancer patients

**Figure 4:**
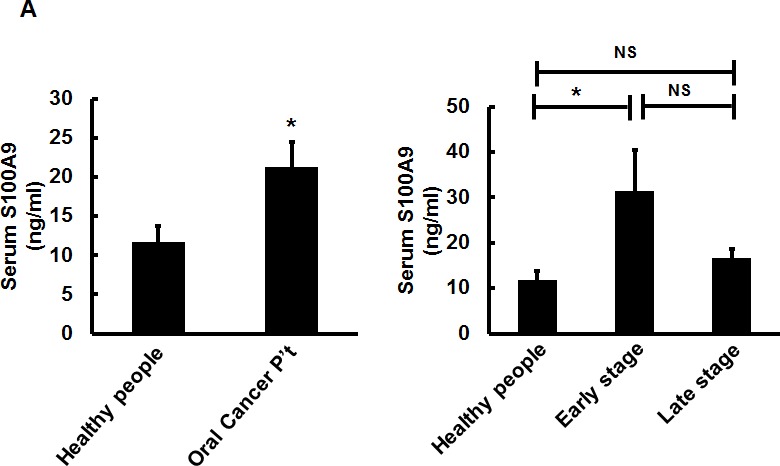

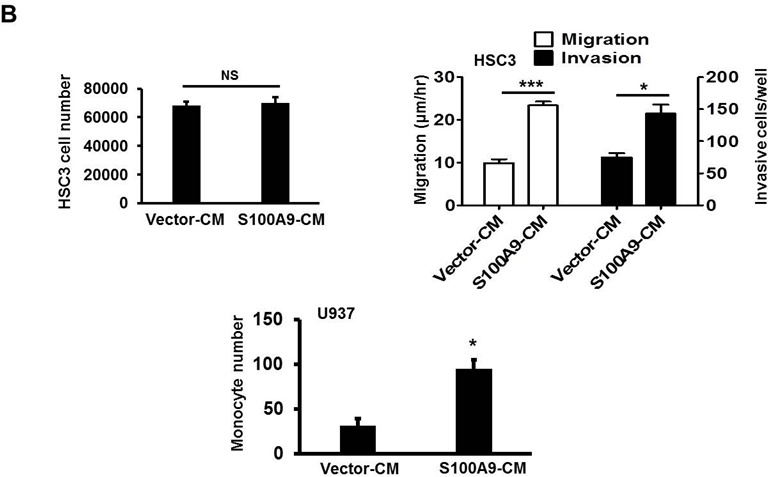

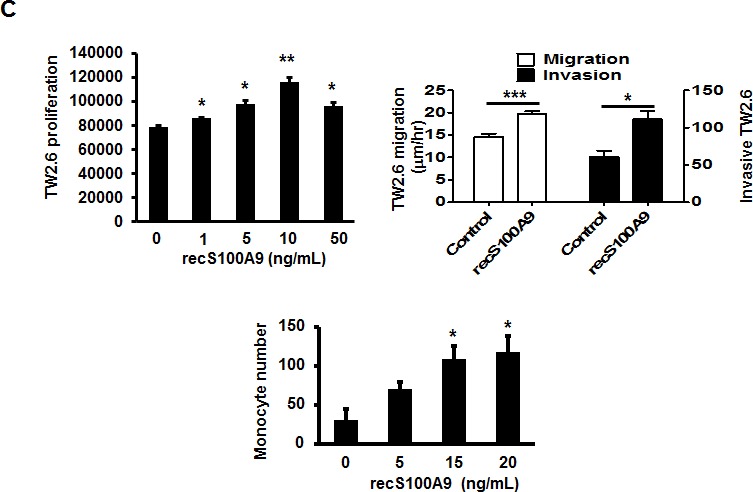

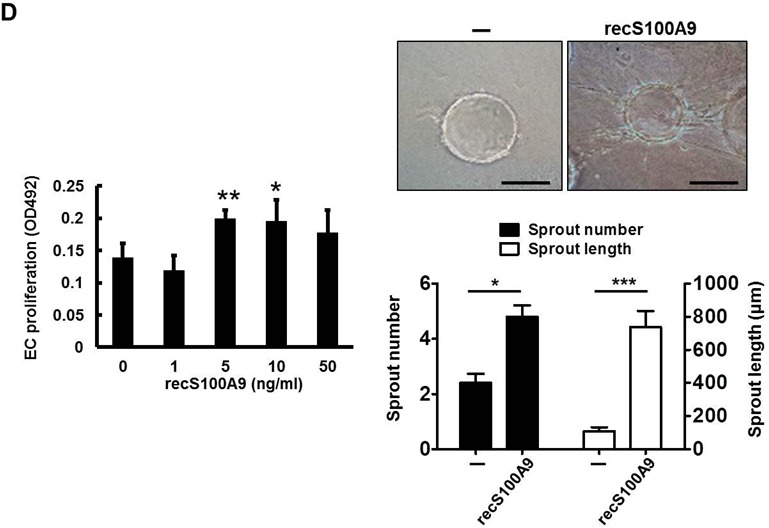

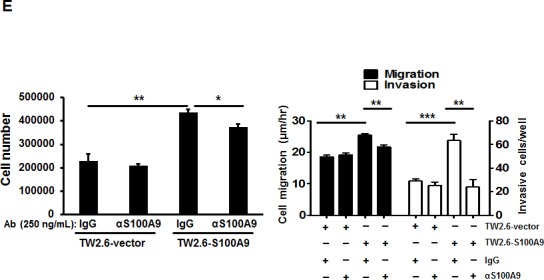

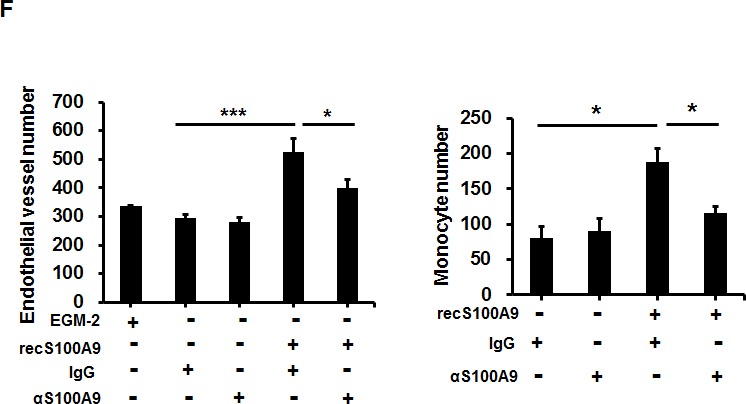
Extracellular S100A9 protein promoted oral cancer migration and invasion, monocytic U937 transendothelial migration, and angiogenesis **A.** S100A9 protein in each control or patient serum was measured three times by ELISA. The concentration of serum S100A9 in 18 age-matched healthy control or 73 oral cancer patients with 23 in early stages and 50 in late stages was expressed as mean ± SEM. *p < 0.05; NS, not significant *versus* healthy volunteers. **B.** Top, following treatment of HSC-3 with vector- or S100A9-CM from TW-2.6 cells for the indicated time, cell proliferation was measured by cell enumeration. Cell migration and invasion abilities of the indicated cells were, respectively, measured by wound healing and cell invasion assays. Data are mean ± SEM. Bottom, human monocytic U937 cells migration across an endothelial monolayer in response to CM from vector or S100A9-expressing TW-2.6. Data are mean ± SD. **C.** Top, following treatment with recS100A9 protein (1-50 ng/mL), TW-2.6 cell proliferation was enumerated and expressed as mean ± SD (Left). The migration and invasion abilities of TW-2.6 cells treated for the indicated time with recS100A9 protein (15 ng/mL) equivalent to the detected level in CM were measured, respectively, by wound healing and cell invasion assays and expressed as mean ± SEM (Right). Bottom, the number of U937 monocyte migration across an endothelial monolayer in response to recS100A9 protein (0-20 ng/mL) in mean ± SD. **D.** Following treatment of recS1009 protein (1-50 ng/mL), endothelial cell proliferation was measured by MTS kits and expressed as mean ± SD (Left). Endothelial cell spheroids were stimulated with recS100A9 protein (15 ng/mL) to induce angiogenic sprouting into the collagen matrix. The mean number of sprouts/bead and the length of sprouts were microscopically assessed (Right). Scale bar, 50 μm. **E.** Vector or S100A9-expressing TW-2.6 cells were subjected to cell proliferation assay by cell enumeration (Left), wound healing and invasion assays with or without anti-S100A9 antibodies (αS100A9 at 250 ng/mL, Right). Data are mean ± SEM. **F.** Endothelial vessel numbers were measured in the endothelial cells treated with recS100A9 (20 ng/mL) together with IgG or αS100A9 antibodies (Left). Transendothelial monocyte migration in response to recS100A9 protein was measured in the presence of IgG or αS100A9 antibodies (Right).

S100A9 can be secreted into culture medium and body fluid [24]. Elevated plasma S100A9 levels are associated with the severity of inflammatory disorders [15]. In the same line with the increase of S100A9 secretion into conditioned medium (CM) derived from S100A9-expressing cells (Figure S7), there was also a significant increase of mean serum S100A9 protein in a separate cohort of 73 oral cancer patients (Table 2) relative to age-matched healthy volunteers by using ELISA (21.56 ± 3.24 *versus* 11.74 ± 2.01 ng/mL, *p* < 0.05, Figure 4A, Left) However, the significant increase was mainly detected in the 23 early-stage but not in 50 late-stage oral cancer patients (Figure 4A, Right), suggesting a pro-inflammatory role of serum S100A9 in early-stage oral carcinogenesis.

**Table 2:** Clinicopathologic characteristics of the 73 oral cancer patients for ELISA

	Number of cases	% of Total
**Medium age (yr)**		
<49	40	54.8
<49	33	45.2
**Stage**		
I	3	4.1
II	20	27.4
III	16	21.9
IV	34	46.6
**Tumor site**		
Buccal + Tongue	60	82.2
Others	13	17.8
**Differentiation**		
Well	55	75.3
Non-well	18	24.7
**Recurrence**		
No	49	67.1
Yes	24	32.9
**2nd primary**		
No	58	79.5
Yes	15	20.5

### Extracellular S100A9 increased oral cancer cell migration and invasion, monocyte transendothelial migration and angiogenesis

Both S100A9-bearing TW-2.6-CM and recS100A9 protein were used to study the extracellular role of S100A9 in cancer cells and 2 stromal cell types, monocytes and endothelial cells (EC). S100A9-bearing CM promoted HSC-3 cell migration and invasion, but not proliferation (Figure 4B, Top). S100A9 was shown to attract monocyte infiltration into lung adenocarcinoma [25] and enhanced the expression of myeloid markers in S100A9-bearing tumor tissues (Figure 2F). We used transendothelial migration to examine the paracrine effect of S100A9 on the migration of monocytic U937 cells through an endothelial monolayer. S100A9-bearing CM significantly enhanced U937 migration through endothelial monolayer (Figure 4B, Bottom). Oral cancer cells, monocytes and EC were individually treated with recS100A9 protein at the indicated doses for the indicated assays. Exogenous recS100A9 recapitulated most of the *in vitro* findings of using CM on oral cancer cell and monocyte behaviors (Figure 4C).

Although recS100A9 at 1-10 μg/mL also stimulated angiogenesis [26], no studies ever showed that S100A9 protein at patient serum levels in nanograms also modulated the angiogenic activity. To examine the role of the paracrine S100A9 on angiogenesis, we used three angiogenic assays. Exogenous recS100A9 protein at 1-50 ng/mL dose-dependently increased EC proliferation and tube formation (Figures 4D and S8). Since S100A9 knockdown also reduced S100A8 protein expression without affecting its mRNA (Figure S9) and S100A8 might play a distinct role from S100A9 [6, 27], anti-S100A9 antibodies was used to neutralize the activity of S100A9 protein. The addition of anti-S100A9 antibodies into the CM attenuated the paracrine effect mediated by S100A9 on oral cancer cell behaviors, particularly the cell invasion, angiogenesis and transendothelial monocyte migration (Figure 4E–4F). Together, exogenous S100A9 promoted monocyte recruitment and angiogenesis in addition to oral cancer cell migration and invasion.

### S100A9 mediated IL-6 release via the crosstalk of oral cancer cells with monocytes

**Figure 5:**
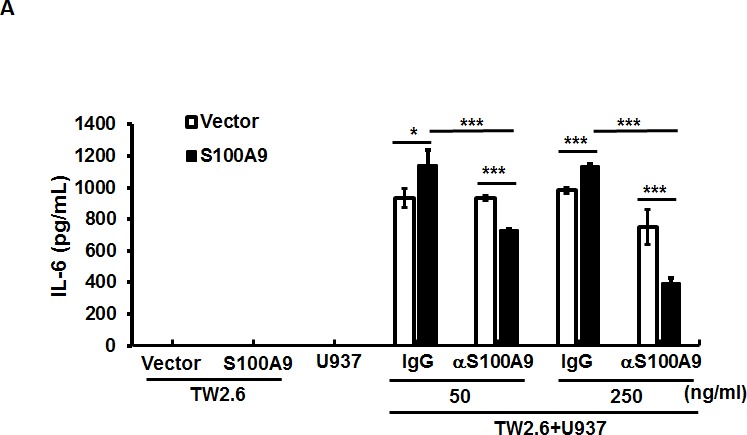

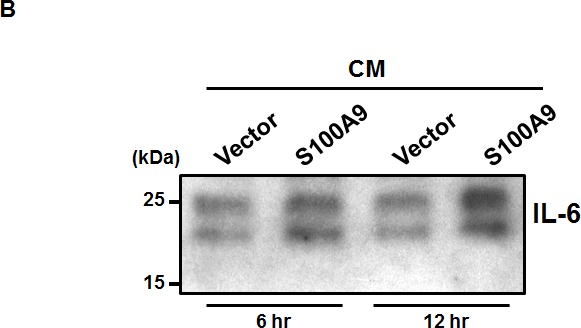

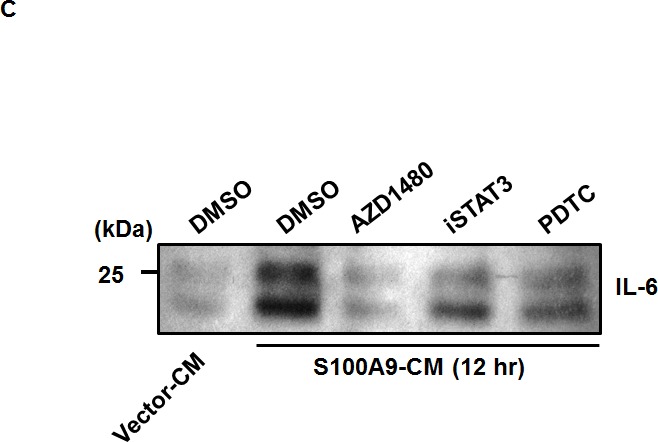
The participation of NF-κB and STAT3 activation in the crosstalk between oral cancer cells and monocytes for IL-6 production **A.** IL-6 concentrations in the CM from mono-culture or co-culture of the indicated TW-2.6 with U937 cells with or without anti-S100A9 antibodies were measured by ELISA and expressed as mean± SD. *p < 0.05, **p < 0.01 or ***p < 0.001 *versus* vector or no treatment. NS, not significant. **B.** Serum-starved U937 cells (10^6^) were treated for 6-12 hr with the indicated CM prior to Western blot analysis. **C.** Serum-deprived U937 cells were pre-incubated for 30 min with 100 μM PDTC, 100 μM iSTAT3, or 2 μM AZD1480 followed by the addition of the indicated CM for 12 hrs prior to protein isolation and Western blot analysis.

Since the expression of IL-6 was predominantly increased in S100A9-bearing xenografts (Figure 2G), we used qRT-PCR to measure if ectopic S100A9 could enhance IL-6 mRNA expression in S100A9-expressing TW-2.6 cultured cells. No significant alterations in IL-6 mRNA expression and the activating phosphorylation of its downstream effectors, NF-κB and STAT-3, were detected in S100A9 cells relative to vector control (Figure S10), indicating a requirement of the interaction between tumor cells and their microenvironment for IL-6 production *in vivo*. To validate this possibility, we measured by ELISA the level of IL-6 protein in the medium of U937 co-cultured with S100A9-expressing TW-2.6 cells. In contrast to a barely detectable IL-6 in mono-culture, IL-6 production was significantly increased in the co-culture medium. The increase of S100A9 expression further enhanced the production. Addition of anti-S100A9 antibodies dose-dependently attenuated both the basal expression and S100A9-mediated increase of IL-6 in the co-culture (Figure 5A).

Due to the lack of the autocrine activation of NF-κB and STAT-3 in the S100A9-expressing oral cancer cells and a crosstalk requirement for IL-6 production, we tested if S100A9-bearing CM from oral cancer TW-2.6 cells would have any effect on the IL-6 production in monocytic U937 cells by using Western blot. As shown in Figure 5B, S100A9-bearing CM time-dependently enhanced the production of IL-6 for as short as 6-hr incubation, indicating an extracellular role of S100A9 in the mediation. NF-κB pathway was often activated in the S100A9-mediated secretion of IL-6 protein [28, 29]. To address the mechanism involved in the mediation, we pre-treated monocytic U937 cells for 30 min with various inhibitors for NF-κB (PDTC), STAT3 (iSTAT3) and its upstream kinase JAK2 (AZD1480) followed by 12-h incubation with Vector or S100A9-CM. The protein lysates were harvested for Western blot analysis of IL-6 protein expression. As shown in Figure 5C, a pharmacological inhibition of NF-κB or STAT-3 pathway attenuated the release of IL-6 protein induced by S100A9-CM. Together, S100A9 increased IL-6 production through the cross-talk of oral cancer cells with monocytes and the activation of NF-κB or STAT-3 participated in the release.

### High S100A9 together with high CD68 in tumor stroma further reduced early-stage patient recurrence-free survival

**Figure 6:**
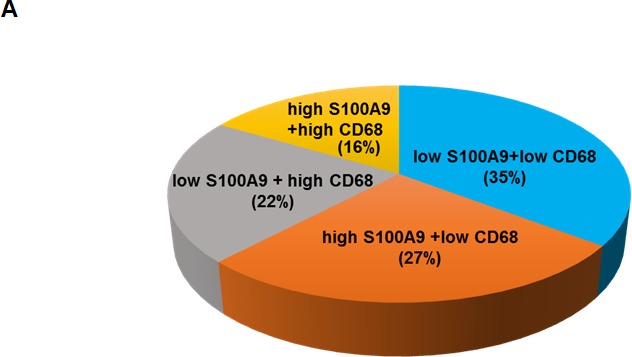

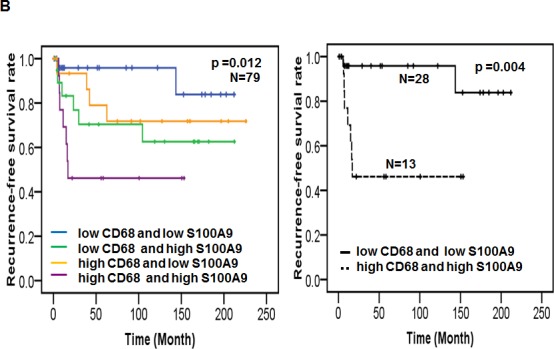

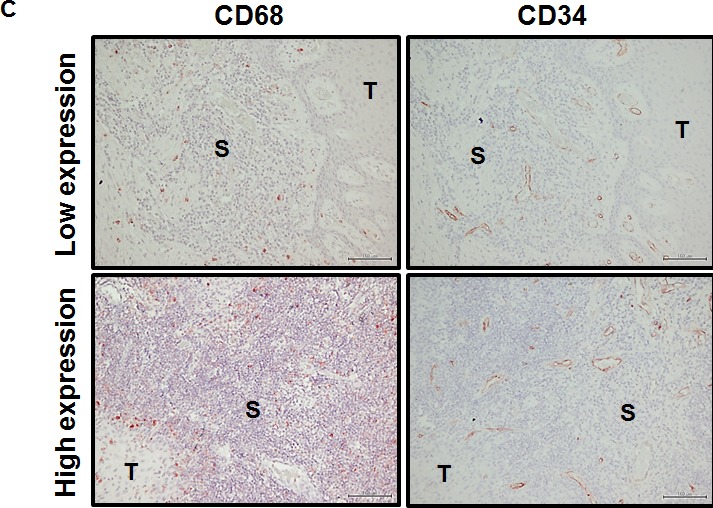

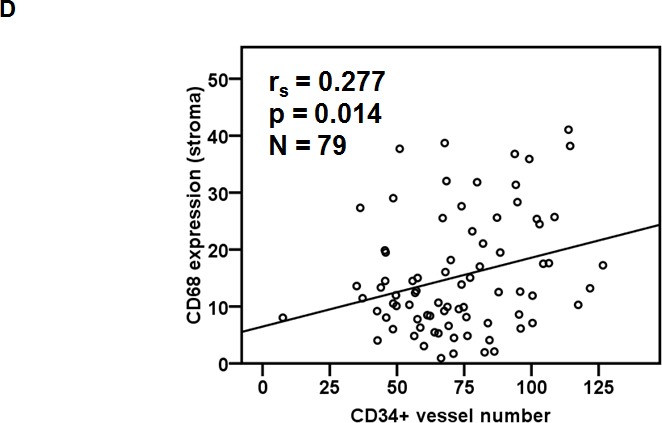
Concomitant high S100A9 with high CD68 protein expression in tumor stroma reduced recurrence-free survival among early-stage oral cancer patients **A.** Seventy-nine early-stage oral cancer patients were divided into 4 groups based on the IHC staining of S100A9 and CD68 in tumor stroma. The pie chart shows the percentage of each group. **B.** Kaplan-Meier analysis showing the relation of recurrence-free survival with the expression of S100A9 and CD68 in stroma. High stromal S100A9 and high CD68 patients had the poorest clinical outcome compared with those with either one high or both low expression. **C.** High or Low expression of both CD68 staining and CD34+ microvessel density in the stroma of two clinical specimens by IHC staining. S, stroma; T, tumor. **D.** Pearson correlation analysis showing a positive correlation of CD34-positive microvessel number with stromal CD68 expression in tumor stroma.

Monocytes serve as precursors for various tissue macrophage and contribute to both protective and pathological immune responses [30]. S100A9 was detected in the CD68+ macrophages of tumor stroma (Figure 3B). Tumor associated macrophages (TAMs) have been shown to promote tumorigenesis and metastasis [31], we used IHC staining to analyze the deregulation impact of CD68 together with S100A9 in tumor stroma on the clinical outcomes of 79 early-stage oral cancer patients. These patients were divided into 4 groups (Figure 6A) for Kaplan-Meier survival analysis based on S100A9 and CD68 staining. Despite lacking a concordant relation of S100A9 and CD68 expression (data not shown), the increase of S100A9 or CD68 in tumor stroma significantly reduced patient recurrence-free survival (Figure 6B, Left, *p* = 0.012). Concomitant high CD68 with high S100A9 expression in stroma had the poorest clinical outcome *versus* those with low expression of both proteins (Figure 6B, Right, *p* = 0.004). Although S100A9 promoted angiogenic activity *in vitro* (Figures 4D and S8), there was no positive association of S100A9 expression with CD34+ microvessel numbers in the stroma (data not shown). Instead, a concordant relation of CD68+ infiltrating cells with the CD34+ vessel number by IHC staining (Figure 6C–6D, r_s_ = 0.277, *p* = 0.014) supported a positive role of infiltrated macrophages in tumor angiogenesis required for tumor growth and metastasis. High expression of both S100A9 and CD68 proteins in tumor stroma was a strong poor prognostic marker for early-stage oral cancer patients.

## DISCUSSION

We have shown the differential abundance of S100A9 expression in both cancer and stroma cells of oral cancer tissues. Early-stage oral cancer patients with the increased S100A9 staining in stroma but not tumor cells had significantly reduced recurrence-free survival when compared with those with low expression. Increased S100A9 expression in oral cancer cells promoted *in vitro* tumor cell migration and invasion, and *in vivo* xenograft tumorigenesis. A predominant increase of human and mouse IL-6 expression, CD31-stained microvessel density and the expression of several myeloid cell markers were detected in S100A9-bearing xenografts. The expression of S100A9 in one stromal component, monoctyes, also enhanced tumor cell aggressiveness. Since S100A9 could be released into the extracellular milieu, we also detected the elevation of S100A9 in early-stage oral cancer patient sera. Extraellular S100A9 exerted a stimulatory effect on oral cancer cell behaviors, monocyte transendothelial migration and angiogenesis. S100A9-mediated increase of IL-6 expression was partly through the cross-talk between cancer and myeloid cells. Taken together, the concomitant increase of both S100A9 and CD68 expression in tumor stroma served as poor prognostic markers for early-stage oral cancer patients.

Despite the presence of S100A8 protein in 3 oral cell lines (Figure 1A), only the increase of S100A9-positive cells in tumor stroma was significantly associated with poor differentiation and shortened recurrence-free survival of early-stage oral cancer patients (Table 1 and Figure 1B–1C). Our data also supports a distinct role of S100A9 from S100A8 in human pathology. Although S100A9 is pro-tumorigenic regardless of its presence in tumor or stroma, the promoting effects were mainly on cancer cell migration and invasion (Figures 2, 3, S5 and S6). The differential response to S100A9-mediated cell proliferation and primary tumor formation in nude mice may be explained by the intrinsically different tumorigenic and metastatic potential of HSC-3 from TW-2.6 oral cancer cells (Figures 2D and S4-5), suggesting an early role of S100A9 in oral carcinogenesis. More studies are needed to examine if tumor S100A9 can enhance HSC3-induced metastasis [32].

S100A9 protein is chemotactic for monocytes [33] and myeloid-derived suppressor cells (MDSCs), a heterogeneous population of immature myeloid cells with tumor-promoting function [34]. The loss of S100A9 gene led to the reduction of MDSC number and lymphoma growth [10]. Due to no availability of murine oral SCC, we were unable to use syngenic mouse model to address the role of S100A9 in the immune-competent mice. Even in the absence of thymus (T cells), six myeloid markers were up-regulated in the nude mice bearing S100A9-xenografts (Figure 2F). Among them, a positive association in the mRNA expression of CD11b and Ly6G, two key markers for granulocytic MDSCs (G-MDSCs), was detected (Figure 2F, Bottom). G-MDSCs were suggested to have a suppressive role for natural killer cell proliferation and activation [35]. Consistent with this notion, we also detected the decrease of natural killer cells (NK1.1) together with reduced CD79a+ B lymphocytes infiltration in these tumors (Figure 2F, Upper Left). The mRNA expression of IL-6, a multi-functional cytokine required for MDSC generation and tumor progression [34], was predominantly increased in S100A9-expressing human tumors and mouse stroma (Figure 2G). The pro-tumor effect exerted by S100A9 in oral cancer was in part via the increase of IL-6 expression and tumor infiltration of G-MDSCs although the involvement of other myeloid cell types and additional cytokines could not be ruled out.

Like most S100 family members, S100A9 was secreted into culture medium and body fluids [24]. Serum S100A9 was increased with the severity of several inflammatory conditions [15]. We also detected the elevation of S100A9 protein in the CM from S100A9-bearing TW-2.6 cells (Figure S7) and an elevated mean serum S100A9 concentration in 73 oral cancer patients relative to controls, with a significant increase in early-stage patients (Figure 4A). S100A9 promotes monocyte extravasation [36]. In addition to stimulating oral cancer cell migration and invasion, S1009-bearing CM or exogenous recS100A9 protein dose-dependently increased the transendothelial migration ability of monocytes (Figure 4B–4C). The stimulatory effect of released S100A9 protein on supporting tumor cell proliferation, migration, and invasion, and monocyte recruitment was attenuated by the addition of anti-S100A9 antibodies (Figure 4E–4F), demonstrating a paracrine effect exerted by S100A9 on oral cancer tissues.

Although the expression of IL-6 mRNA was significantly induced in S100A9 xenograft tissues (Figure 2G), there was no enhancement of IL-6 expression and downstream mediators in S100A9-expressing oral cancer monoculture (Figure S10). The detection of IL-6 increase *in vivo* but not *in vitro* suggests a requirement of the crosstalk between tumor and stroma cells for the induced IL-6 expression in tumors. Co-culture of oral cancer cells with monocytic U937 cells confirmed the increased IL-6 production and the presence of S100A9 enhanced the increase. S100A9 neutralization dose-dependently attenuated the increased IL-6 production in the co-culture medium (Figure 5A). The activation of both NF-κB and STAT3 participated in the mediation (Figure 5C). The extent of IL-6 increase induced by S100A9 in the co-culture medium was, however, not as significant as that in xenograft tumors (Figure 2G), suggesting the involvement of additional cell types in the production of IL-6 *in vivo* (Figure 3B).

S100A9 was previously detected in the myeloid cells of both acute and chronic inflammatory diseases [37], especially in circulating neutrophils and monocytes but absent in lymphocytes [38]. Myeloid cells are precursor cells for monocytes, macrophages and neutrophils. In this report, S100A9 was detected in the stromal cells including CD11b+ monocytes, CD15+ neutrophils or CD68+ macrophages (Figure 3B). Extravasated monocytes have the potential to differentiate into TAMs as they are in tumor tissues. Several clinical studies including oral cancer have shown a strong correlation of high TAM number with increased microvessel density, indicating a positive role of TAMs on tumor angiogenesis [39, 40]. Macrophages are markedly positive for CD68. Early-stage oral cancer patients with both high S100A9 and high CD68 had the poorest clinical outcome relative to those with high expression of either protein alone, or those with low expression of both proteins (Figure 4D). Although S100A9 potently induced angiogenesis *in vitro* (Figures 4D and S8), no positive association of S100A9 with CD34, an endothelial marker, was detected in these clinical specimens. Instead, CD34+ vessel number was positively associated with the presence of CD68 in tumor stroma (Figure 4E–4F), furthering the notion that S100A9 promotes angiogenesis *in vivo* through increased macrophage recruitment.

In summary, S100A9 exerted pro-tumor effects through not only autocrine effect on cancer cells but also paracrine effect on stromal cells, including inflammation promotion and angiogenic activation. This study provides insights into the selection for early-stage oral cancer patients with concomitant high S100A9 with high CD68 in the stroma that most likely benefit from aggressive treatments. A S100A9-binding small molecule (ABR-215050) that inhibits the interaction between S100A9 and TLR4, the major receptor required for chemically-induced oral carcinogenesis [41], is presently in Phase III clinical trial for treating prostate cancer [42]. Besides supporting the possibility of using this compound for treating oral cancer, the neutralization of IL-6 induced by S100A9 may also function as potent anti-neoplastic agents in oral carcinogenesis, in which the stromal S100A9 plays a significant role in regulating tumor growth and metastasis.

## MATERIALS AND METHODS

### Materials

Keratinocyte serum-free medium (KSFM), all the other medium power, Trizol reagent, Lipofectamine 2000, OPTI-MEM, anti-Myc epitope antibodies and zeocin were from Life Technologies (Grand Island, NY., USA). Endothelial growth medium 2 (EGM2) for growing endothelial cells was from Lonza Inc. (Allendale, NJ, USA). All the chemicals, anti-His tag antibodies and Wortmanin were from Sigma-Aldrich Co. (St. Louis, MO, USA). CellTiter 96^®^ AQueous One Solution (MTS kit) was from Promega Corp (Madison, WI, USA). pLKO_AS2.zeo was from National RNAi Core facility in Academia Sinica, Taiwan. Anti-CD15, anti-CD68, and anti-CD11b antibodies were from Leica Biosciences (Richmond, Illinois, USA). S100A9 antibodies and STAT3 inhibitor VI (iSTAT3) were from Santa Cruz Biotechnology (Dallas, Texas, USA). Anti-human CD34 antibody was from DakoCytomation Denmark A/S (Copenhagen, Denmark). Anti-S100A8 antibodies, human recombinant S100A9 (recS100A9) protein and anti-CD31 antibodies were from Abcam (Cambridge, MA, USA). Matrigel was from BD Biosciences (San Jose, CA, USA). Pyrrolidine dithiocarbamate (PDTC) was from Tocris Bioscience (Bristol, UK). AZD1480 was from Selleckchem (Houston, TX, USA).

### Patient tissue and serum specimens

Paraffin-embedded blocks of 79 early-stage oral cancer specimens including stages I and II were obtained with informed consent. This study population included 71 males and 8 females with a median age of 48 years (Table 1). Peripheral venous blood (5 ml per patient) was obtained from a separate cohort of 73 oral cancer patients (Table 2) consisting of 23 in early-stage and 50 in late-stage with a median age of 49 years (ranging 36-75 years) and 18 age-matched healthy volunteers with informed consent. Individual serum was prepared from blood by centrifugation at 3000 rpm for 15 min at 4°C and stored at −80°C until analysis. Their use and protocols for this study were approved by the Institutional Review Board at National Cheng Kung University.

### IHC staining

Patient tissue sections (5 μm thickness) were deparaffinized through a gradient alcohol and xylene. Hematoxylin and eosin staining was used to confirm the original histopathological diagnosis. Following quenching endogenous peroxidase by hydrogen peroxide, an antigen retrieval method was employed to enhance the immunodetection before antibody incubation. Consecutive tissue sections from the same patient were individually incubated overnight with the indicated antibodies at 4°C followed by incubation with the secondary antibody. The immunocomplexes were detected by a standard biotinylated streptavidin-alkaline phosphatase-based kit (Dako, Hamburg, Germany) and AEC Single Solution (Invitrogen, Grand Island, NY, USA). The same sections were counter-stained with 1% hematoxylin. The stromal S100A9 and CD68 staining in five to 10 random fields (400X magnification) were quantified and digitalized by HistoQuest (Tissue Gnostics, Vienna, Austria). The patients were divided into two groups. High group include the patients with greater than and low with equal to or less than the mean or median percentage of cell population stained with both AEC and hematoxylin stain. We counted CD34+ microvessels in 5 random fields (200X magnification) as described [43] and calculated the mean CD34-positive vessel number in each tumor section for statistical analysis.

### Statistical analysis

We used Pearson χ2 test to compare high and low S100A9 expression in relation to clinicopathologic characteristics of 79 early-stage oral cancer (Table 1). The Kaplan-Meier method and log-rank test were used to compare the overall and recurrence-free survival in the patients with high and low S100A9 groups. Two to three biological repeats for cell-based studies and eight mice per group were statistically analyzed by Two-tailed Student’s *T*-test. Data represent meanSEM. *P* < 0.05 was considered as statistical significance.

### Cell culture

Normal oral keratinocytes (NOK) were cultured in KSFM. Displastic oral keratinocytes (DOK) and six oral cancer cell lines, CAL-27, OC-2, OC-3, OEC-M1, HSC-3 and TW-2.6, were maintained as described [32, 44, 45]. Human 293T and monocytic U937 ells were maintained as described by American Tissue Culture Collection. Human immortalized microvascular endothelial cell line HMEC-1 [46] was propagated in EGM2.

### Western blot analysis

Cells were lysed in boiling SDS lysis buffer. The protein concentration was measured by Bradford protein assay. Equal amounts of total proteins were fractionated by SDS-PAGE and blotted onto polyvinylidene difluoride membrane. The protein blots were hybridized with the indicated primary and then secondary antibodies in 5% non-fat milk, followed by detection with Immobilon Western system (Millipore Corp., Billerica, MA).

### Construction of human S100A9 expression vectors

The S100A9 coding sequence-bearing PCR products spanning from nucleotides 13 to 385 (S100A9, Genbank accession no. NM_002965) were cloned into a mammalian expression vector pcDNA3.1(−)/Myc-His A (Invitrogen). The Myc/His-tagged S100A9 cDNA fragment was sequence-verified and then cloned into pLKO-AS2.zeo lentiviral vector for stable expression in the cells.

### Establishment of ectopic S100A9 expressing stable clones

Vector and S100A9-bearing lentiviruses were prepared from transfection of human 293T cells, respectively, with pLKO-AS2.zeo-S100A9 plasmids or pLKO-AS2.zeo vector using Lipofectamine 2000. Viral particles in the medium were collected 48 hrs after transfection. Following infection with the vector or S100A9-bearing lentiviruses, stable S100A9-expressing or vector control cells were enriched with zeocin (400 μg/ml) for 1-2 weeks before the indicated experiments.

### Cell proliferation assay

Two different assays, cell enumeration and OD_492_ measurement by MTS kits, were used for measuring cell proliferation. For cell enumeration, the indicated cells were seeded in triplicate at 10-20% confluence in 24-well plates. Cells were harvested for viable cell count by trypan blue exclusion on daily basis for 4 days after seeding. For OD_492_ measurement, the indicated cells were seeded in quadruplicate in 96-well plates and subjected to growth in a CO_2_ incubator for 2 days prior to the use of MTS kits to measure cell proliferation. This experiment was independently repeated for 3 times. Data are mean±SD.

### Wound healing

The indicated cells were seeded in duplicate at 90% confluence in 6-well plates pre-coated with 5 μg/ml collagen. Once the cells reached confluence, the cells were treated with mitomycin C (2 μg/ml) to stop cell proliferation for 24 hrs. We used 200-μl loading tips to generate wounds on the monolayer. Cell migration was monitored and photographed at 0 - 48 hrs post-wounding depending on the cell types. The mean distance of ten wound width along the wound before and after migration was calculated. The migration rate was the cell migration distance per hr and expressed as Mean± SEM. This experiment was independently repeated 3 times.

### Cell invasion assay

Following coating the upper wells with Matrigel (100 μg/well) for 2 hrs at 37°C, each lower well in Transwell units was filled with 500 μl of the appropriate growth medium. Serum-starved cells in 250 μl of serum-free medium were added in triplicate to the upper well followed by incubation at 37°C for 24 hrs. Following the removal of the un-migrated cells on the top side of the membrane, the migrated cells on the bottom side of the membrane were fixed and stained with 0.1% crystal violet for 15 minutes at room temperature prior to the enumeration under microscopy. The experiment was independently repeated 3 times. Data represent mean±SEM.

### Immunofluorescence staining

Following the paraffin removal from paraffin-embedded tissue section, the section was permeabilized with 0.2% Triton X-100 in phosphate-based saline (PBS) for 45 minutes at room temperature. Tissue sections were blocked with 2% FBS and 0.1% bovine serum albumin in PBS and hybridized with the indicated primary and fluorescence-conjugated secondary antibodies. Stained tissues were observed by Olympus FV1000MPE multi-photon laser scanning microscope.

### Xenograft transplantation and immunohistochemistry

Male BALB/cAnN.Cg-*Foxnl^nu^*/CrlNarl mice (6-8 weeks old) were purchased from National Laboratory Animal Center. Ectopic S100A9-expressing TW-2.6 cells (2×10^6^ cells) or vector control cells together with 50 μg Matrigel (BD Sciences, San Jose, CA, USA) were subcutaneously injected into the flanks of nude mice (8 mice in each group). One week after injection, tumor size was measured every 2 days for 53 days. Tumor tissues were harvested at the endpoint for IHC staining and total RNA/protein isolation following weight measurement. The animal use for this protocol was reviewed and approved by the Institutional Animal Care and Use Committee. The numbers of Ki67-positive nuclei and -negative nuclei as well as CD31+ microvessels in 5 random fields (200X magnification) were counted by using Image J software. The percentage of Ki67-positive nuclei (mean ± SEM) in total counted nuclei of each tumor tissue was expressed as mean ± SEM.

### Co-culturing oral cancer cells with monocytes

To differentiate oral cancer cells from monocytic U937 cells, we infected the indicated oral cancer cells with Fu-mCherry-bearing lentiviruses. The mCherry-expressing oral cancer cells were co-cultured with S100A9-overexpressing or vector control U937 cells in 2:1 ratio prior to cell proliferation assay. The mCherry fluorescent signal was detected by the SpectraMax M3 Multi-Mode Microplate Reader (Molecular Devices, Sunnyvale, CA, USA). A reference standard curve for converting sample fluorescence values into cell numbers was used. Data represent mean ± SD. For the migration and invasion assays, the serum-starved mCherry-expressing oral cancer cells (10^5^ cells per well) was seeded in triplicate onto upper wells coated with or without Matrigel of 24-well Transwell plates while S100A9- or vector-U937 cells (5×10^4^ cells/well) were seeded in the lower wells. The number of migratory or invasive oral cancer cells on the bottom of the membrane was stained and counted as described in the cell migration and invasion assays.

### CM preparation

The indicated cells or co-cultures were seeded at 80% confluence in 10 cm dish. Cells were refed with 6 ml of serum-free M199 one day after seeding. The CM was harvested at 24 hour after incubation and centrifuged at 3000 rpm to remove the cell debris. The supernatant was concentrated by using Vivaspin 6 columns (5 kDa MWCO, GE Healthware Life Science, Piscataway, NJ, USA).

### Measuring S100A9 or IL-6 concentration by ELISA

The concentrations of S100A9 protein in CM from mono-culture or co-culture and those in patient sera were measured in triplicate by CircuLex S100A9/MRP14 ELISA kit (Abnova Corp., New Taipei City, Taiwan). The detection sensitivity for this kit was 6.55-3200 pg/mL without the cross-reactivity to the other S100 proteins. The IL-6 level in the CM and patient serum was individually quantified in triplicate by using the Quantikine human ELISA assay (R&D systems, Minneapolis, MN, USA). We calculated the serum concentration of S100A9 or IL-6 based on serial dilutions of a recombinant protein at known concentrations.

### Antibody neutralization

To study the S100A9-mediated effect, anti-S100A9 antibodies at the indicated concentrations were added onto the treatment medium prior to its use for treating the indicated cells for cell proliferation, migration, invasion, angiogenic assays, monocyte transendothelial migration and total RNA isolation.

### Quantitative RT-PCR (qRT-PCR)

Total RNA was isolated by using TriZol reagents from the indicated cells or snap-frozen S100A9-bearing xenograft tissues. One μg RNA was reverse-transcribed into cDNA using High Capacity cDNA Reverse Transcription Kit (Applied Biosystems, Foster City, CA, USA). We amplified cDNA samples by using the SYBR Green PCR Master Mix (Roche, West Sussex, UK) and determined the cycle threshold (Ct), the fractional cycle number at which the amount of an amplified target reaching a fixed threshold. The mRNA expression of the indicated genes in triplicates was calculated by using 2^−ΔCt^ (ΔCt = Ct^target gene^-Ct^18S rRNA)^. The primers were listed at Table S1.

### Transendothelial migration of monocytes

HMEC-1 cells (150,000 cells/well) seeded in triplicate on collagen-coated Millicell culture inserts (8μm pore size, Merck Millipore Ltd.) were grown to confluence for 3 days. Monocytic U937 cells (10,000 cells per well) were seeded onto the inserts with TNF-α-activated or untreated endothelial monolayer (a negative control) and allowed to migrate for 24 hrs to the bottom wells. The living cells in the bottom wells were counted by trypan blue exclusion assays under a light microscope.

### Angiogenic assays

HMEC-1 cells (5000 per well) in gelatin-coated 96-well plates were starved for 12 hrs with M199 containing 1% FBS and 0.1% bovine serum albumin. Starved cells were seeded in triplicate and treated for 2 days with the indicated CM, M199 or M199 with recombinant S100A9 protein (0-50 ng/mL) in the presence of 2% FBS followed by using MTS kits for measuring endothelial cell proliferation. Two tube formation methods were used. One was the microcarrier bead sprouting assay as described [47]. Following coating Cytodex 3 beads with HMEC-1 for 4 days, EC-covered beads were seeded on top of fibrin gel (10 mg/ml). Microphotographs of vessel outgrowth on beads were taken at day 4-8 after recS100A9 treatment (15 ng/mL). Vessel numbers and vessel length per beads were quantified by Image J (National Institutes of Health, USA). The other was based on tube-like structure formation on Matrigel. The same cells (2×10^4^ per well) were seeded in duplicate onto 48-well culture dishes coated with 100 μl of Matrigel (13.4 mg/mL) and subjected to recS100A9 treatment (0-25 ng/mL). Tube formation was photographed at 3 h post-seeding with an inverted Olympus CKX31 phase-contrast microscope (Tokyo, Japan). The branch point number in 4 random high-power fields (40X magnification) was quantified by the imaging software developed by Dr YN Sun at National Cheng Kung University.

### IL-6 expression in the U937 cells treated with the indicated CM in the presence of various kinase inhibitors

Serum-starved U937 cells (10^6^) were treated for 6-12 hrs with indicated CM prior to protein harvest for Western blot analysis of IL-6 protein expression. For the pharmacological inhibition, 10^6^ serum-starved U937 cells were pre-treated for 30 min with 100 μM PDTC (NF-kB inhibitor), 100 μM iSTAT3 (STAT-3 inhibitor), 2 μM AZD1480 (JAK2 inhibitor) followed by CM treatment for 12 hrs prior to protein isolation.

## SUPPLEMENTARY MATERIAL FIGURES AND TABLE


