# Supplementary material for: Elevated S100A9 expression in tumor stroma functions as an early recurrence marker for early-stage oral cancer patients through increased tumor cell invasion, angiogenesis, macrophage recruitment and interleukin-6 production
Source: Oncotarget. 2015 Jul 22;6(29):28401–24. doi: 10.18632/oncotarget.4951 (PMC4695068; doi:10.18632/oncotarget.4951)
Supplement: Supplementary file 1 [file oncotarget-06-28401-s001.pdf]

Elevated S100A9 expression in tumor stroma functions as an early recurrence marker for early-stage oral cancer patients through increased tumor cell invasion, angiogenesis, macrophage recruitment and interleukin-6 production

Supplementary Material

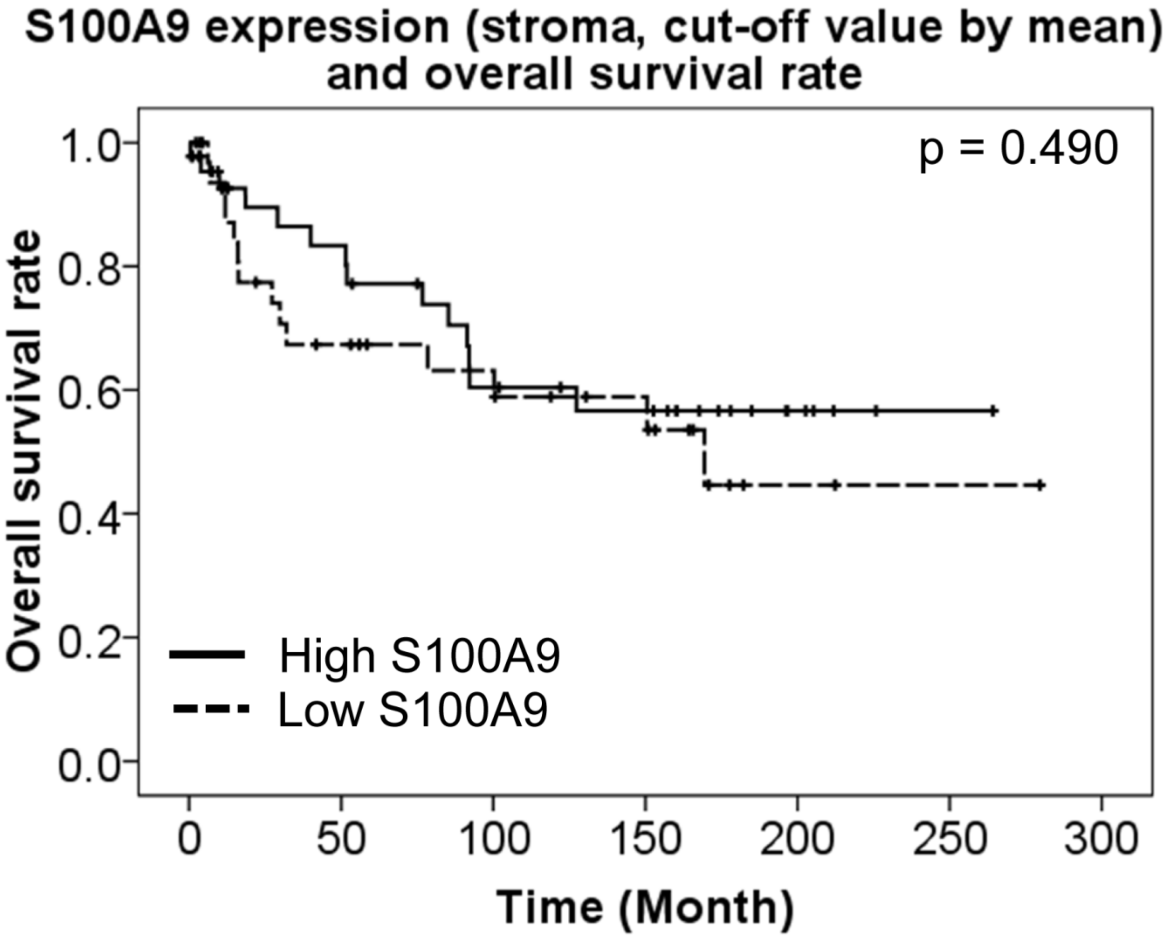

Figure S1 Stroma S100A9 deregulation had no impact on the overall survival of early-stage oral cancer patients. All the 79 patients were divided into two groups based on the mean expression of S100A9 in the tumor stroma. Kaplan-Meier analysis and Log-rank test were used for statistical comparison of the overall survival between the patient groups. High, greater than mean. Low, equal to or less than mean or median.

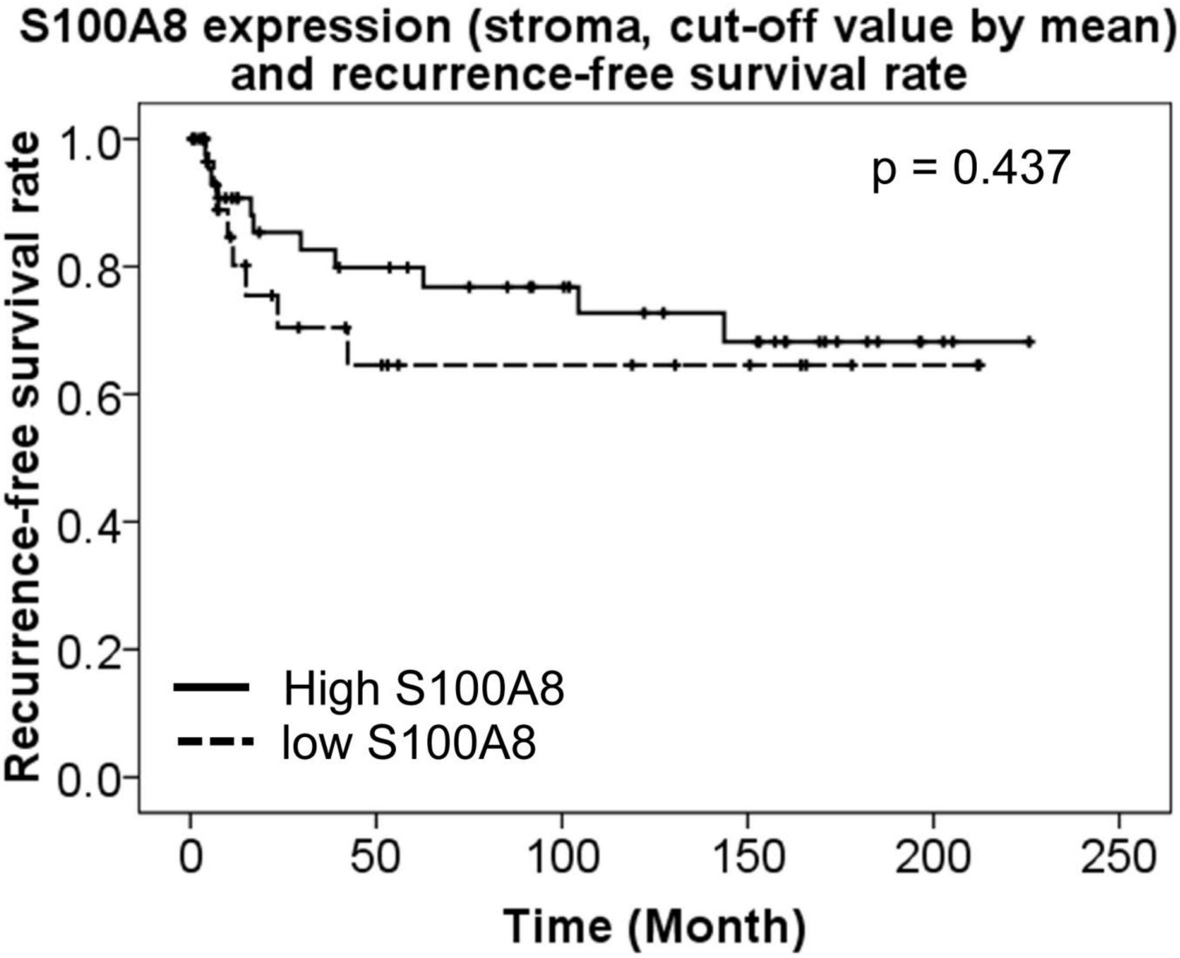

Figure S2 High stroma S100A8 expression had no effect on the patient recurrence-free survival. All the 79 patients were divided into two groups based on the mean expression of S100A8 in the tumor stroma. Kaplan-Meier analysis and Log-rank test were used for statistical comparison of the recurrence-free survival between the patient groups. High, greater than mean. Low, equal to or less than mean or median.

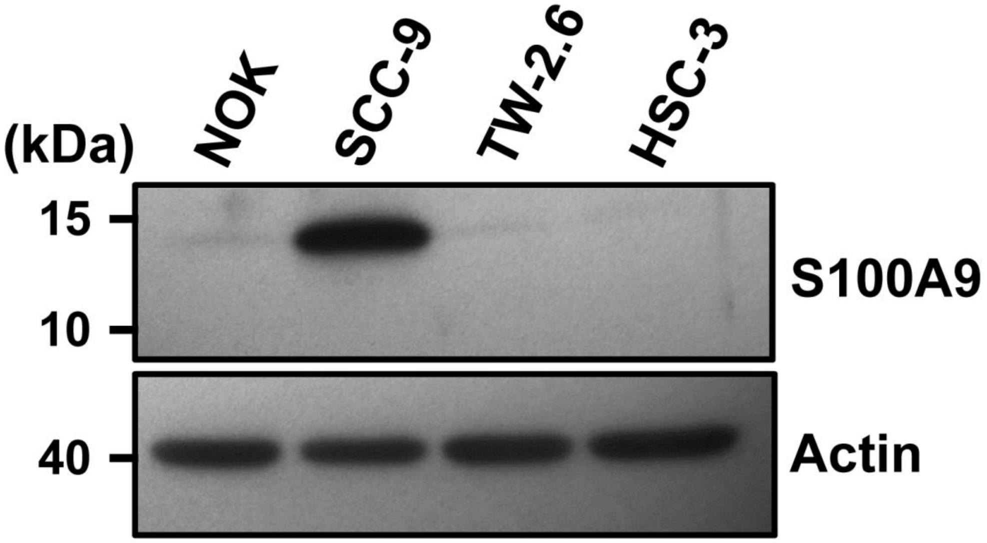

Figure S3 Both TW-2.6 and HSC-3 have comparably low S100A9 expression in the cells. The expression of S100A9 expression in NOK, SCC-9, TW-2.6 and HSC-3 cell lines was detected by Western blot analysis. Actin is a loading control.

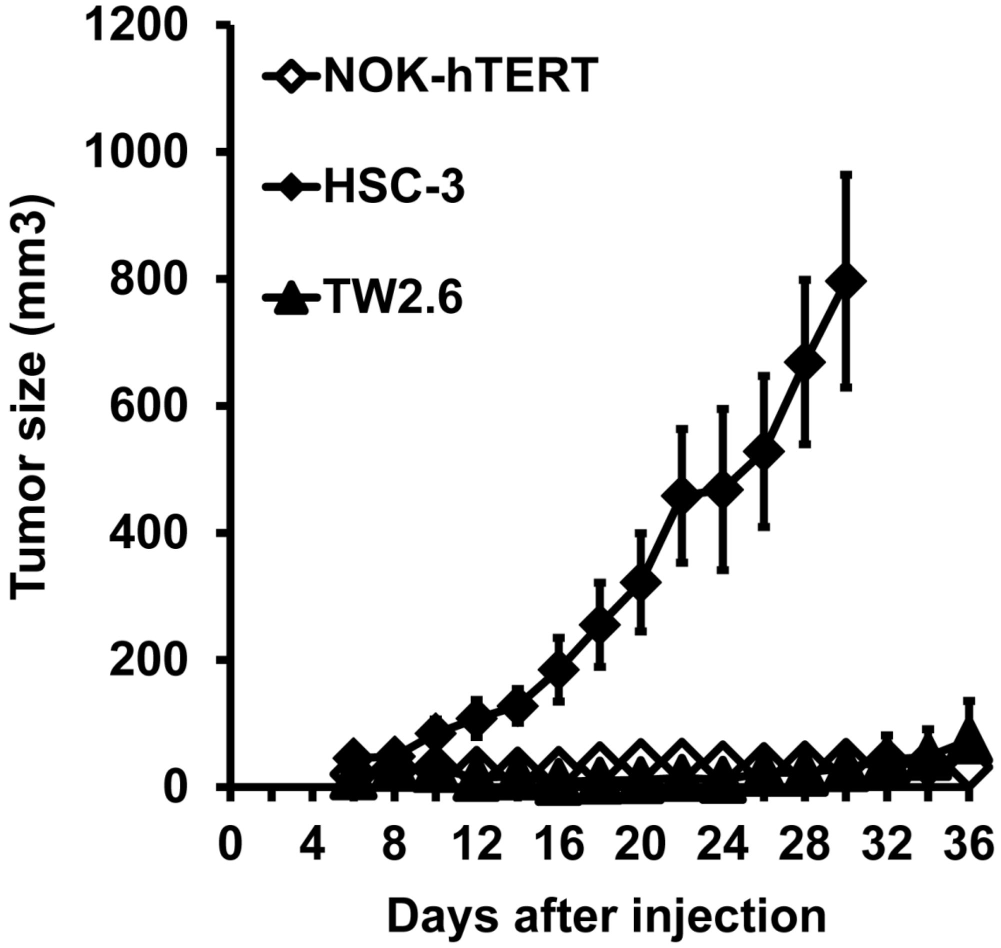

Figure S4 HSC-3 cells was highly tumorigenic than TW-2.6 in nude mice. The indicated cells, immortalized NOK-hTERT, TW-2.6 or HSC-3 ($2x10^6$ cells each), together with 50 μg Matrigel were subcutaneously injected into the flank of nude mice (5 mice in each group). One week after injection, tumor size was measured every 2 days for 36 days.

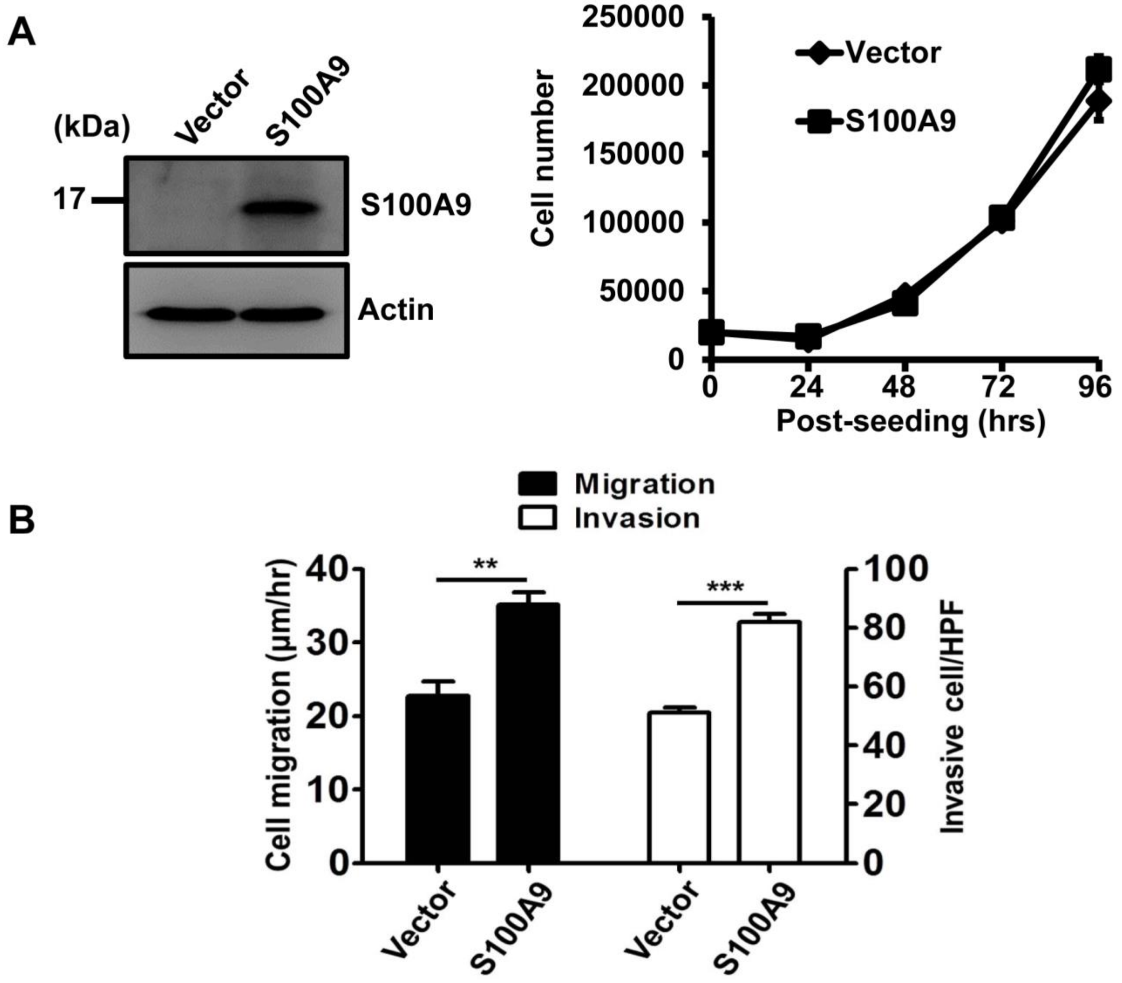
A
(kDa)
Vector
S100A9
17
S100A9
Actin
250000
200000
150000
100000
50000
0
Cell number
Vector
S100A9
0
24
48
72
96
Post-seeding (hrs)
B
Migration
Invasion
Cell migration (μm/hr)
40
30
20
10
0
**
***
100
80
60
40
20
0
Invasive cell/HPF
Vector
S100A9
Vector
S100A9

C

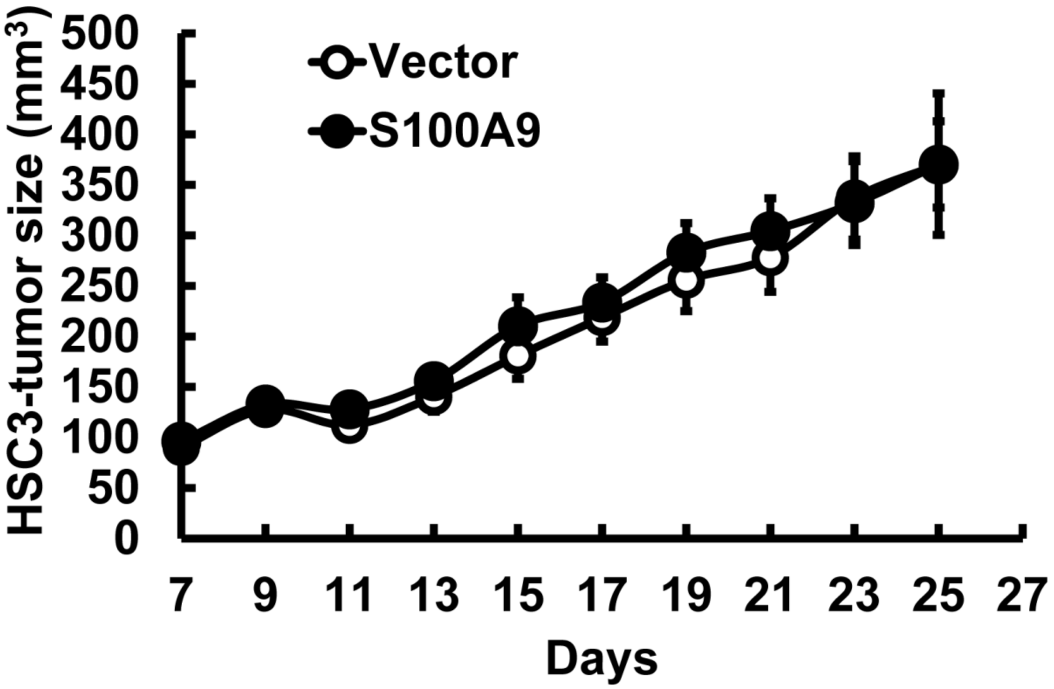

Figure S5 Ectopic S100A9 expression increased HSC-3 oral cancer cell migration and invasion but not proliferation. (A) HSC-3 cells were infected with lentiviruses bearing empty vector or human S100A9 expression. S100A9 expression was measured by Western Blot analysis (Top left). The indicated viable cells was numerated on daily basis for 4 days for cell proliferation assay (Top right). (B) cell migration and invasion abilities of the indicated cells were, respectively, measured in triplicate by wound healing and cell invasion assays and were expressed as mean± SEM. ** $p<0.01$ versus vector control. (C) S100A9- or vector- expressing HSC-3 cells were subcutaneously injected into male nude mice (8 mice for each group). Tumor sizes were measured every 2 days for 25 days due to high tumorigenic potential of HSC-3 cells. In the same line of no stimulatory effect on *in vitro* cell proliferation, ectopic S100A9 expression had no stimulatory effect on HSC-3 tumor xenografts even at 25 days post-injection.

A

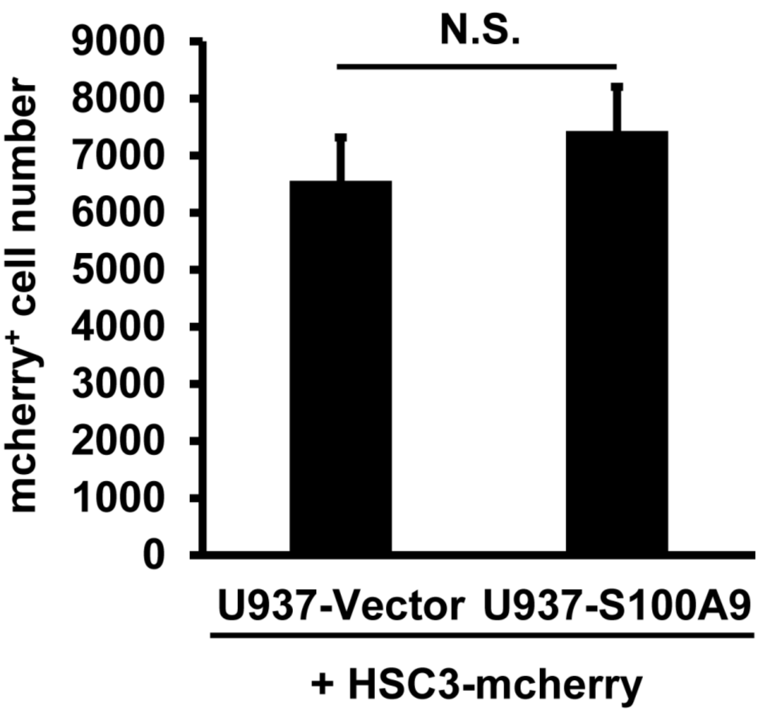
N.S.
mcherry+ cell number
9000
8000
7000
6000
5000
4000
3000
2000
1000
0
U937-Vector
U937-S100A9
+ HSC3-mcherry

B

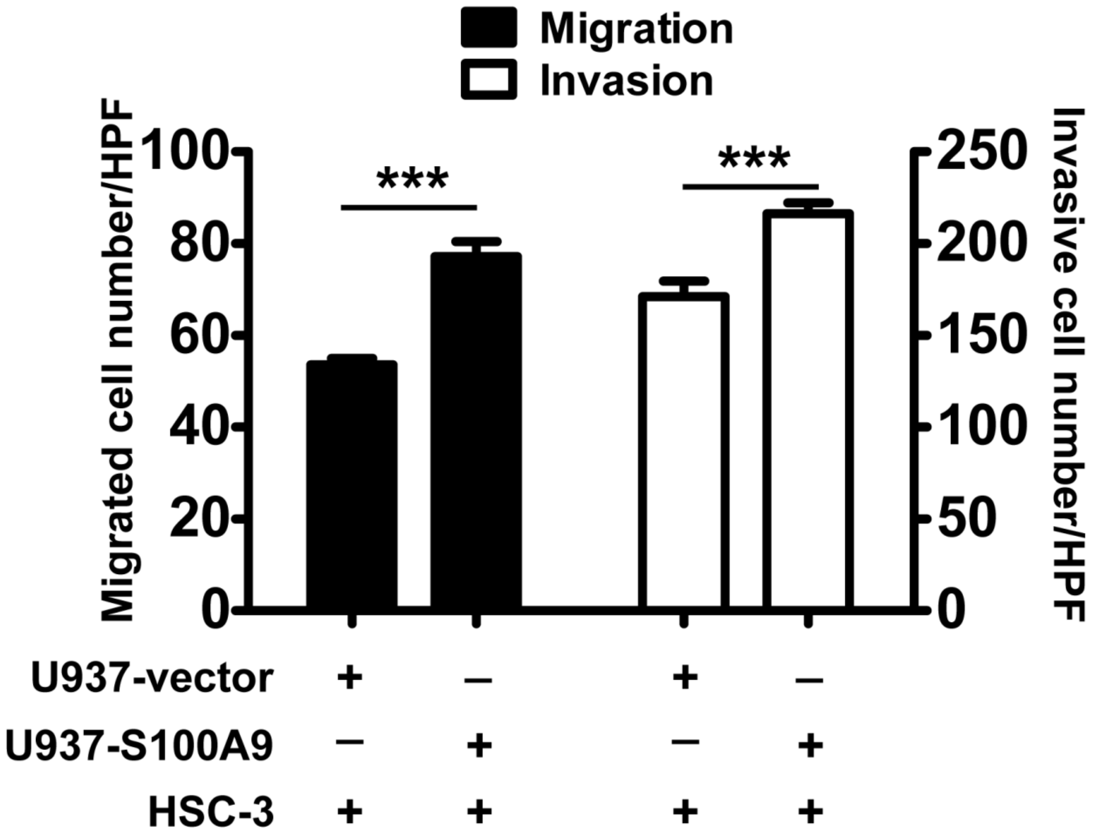

Figure S6 S100A9-expressing U937 cells increased HSC-3 cell migration and invasion but not proliferation. (A) HSC-3-mCherry expressing cells were incubated with the indicated U937 cells for 48 hours. The number of mCherry-positive HSC-3 cancer cells was measured by a SpectraMAX M3 microplate reader. Data are mean ± SD. (B) Cell migration and invasion abilities of HSC-3 cells co-cultured with indicated U937 cells were measured by using Transwell plates. TW-2.6 cells seeded in the inserts were incubated with the indicated U937 cell clones in the bottom wells for 24 hours. Data are mean ± SEM. N.S., not significant. * p<0.05 or *** p<0.001 versus vector.

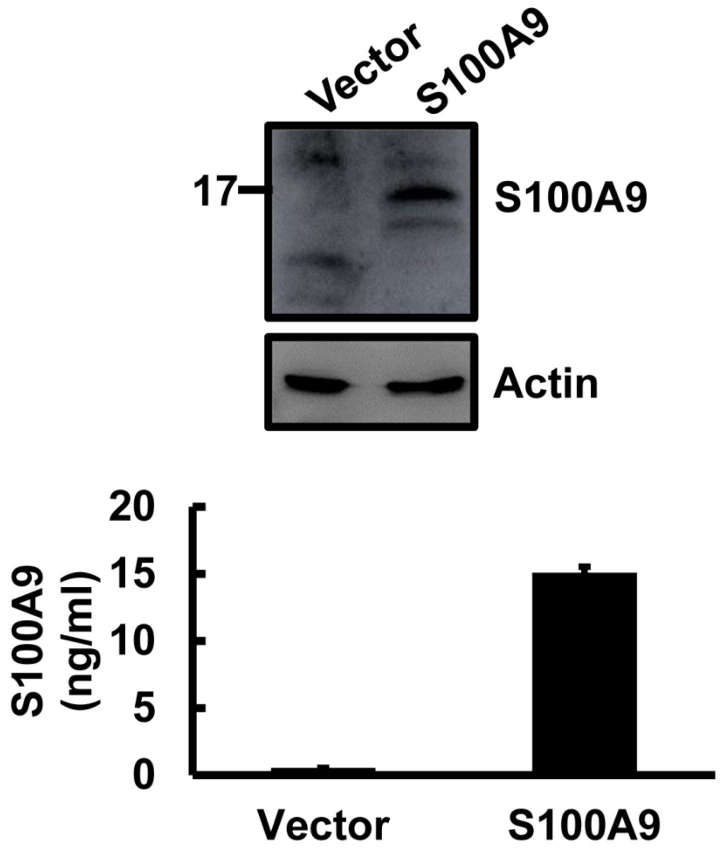

Figure S7 The release of S100A9 in the CM of S100A9-bearing oral cancer cells

The expression of S100A9 protein in the CM derived from TW-2.6-vector or -S100A9 was, respectively, analyzed by Western Blot (Top) and ELISA analyses (Bottom). ELISA data are mean±SEM of 3 repeats.

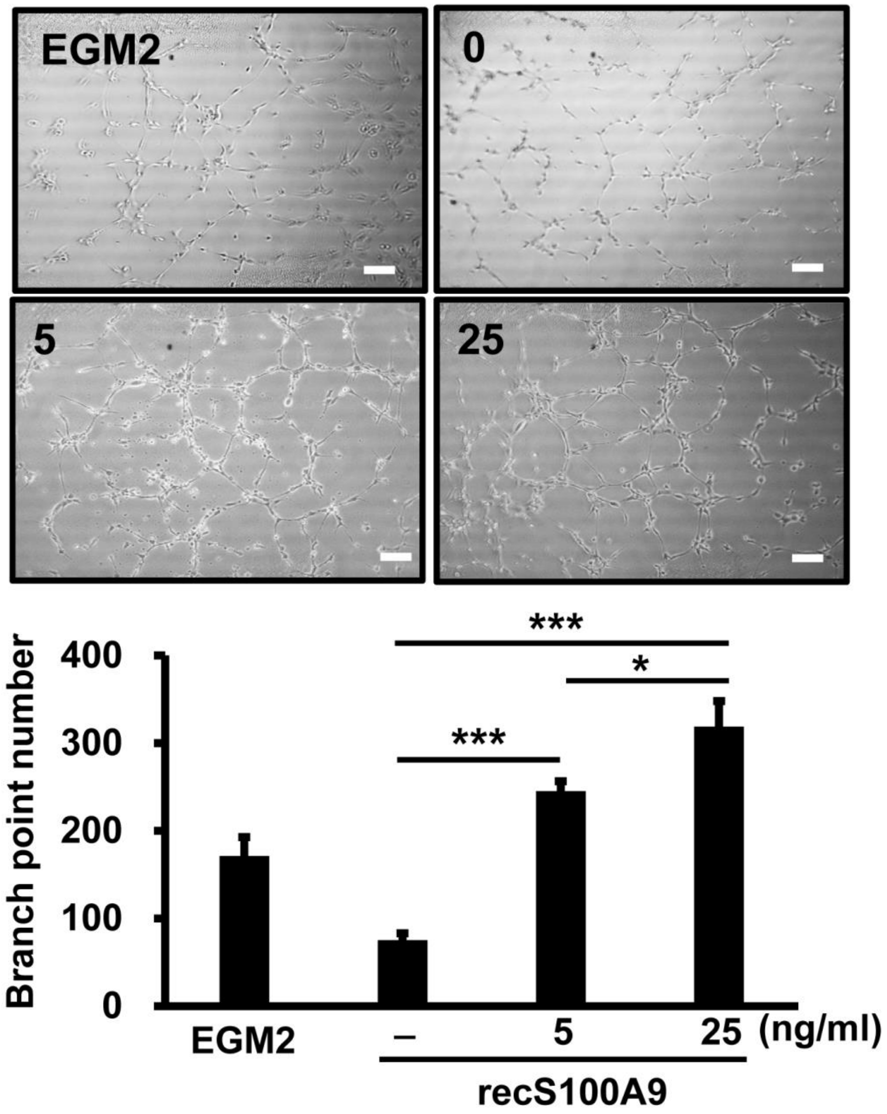

Figure S8 recS100A9 dose-dependently increased endothelial tube formation on Matrigel. Endothelial cells were grown on Matrigel-coated plates in vehicle only, different dosages of S100A9 protein (5-25 ng/mL) and EGM-2 (positive control). The branch point numbers in 4 random 200X fields were expressed as mean ± SD (N=2). Scale bar, 100 μm. * $p<0.05$ or *** $p<0.001$ versus no treatment.

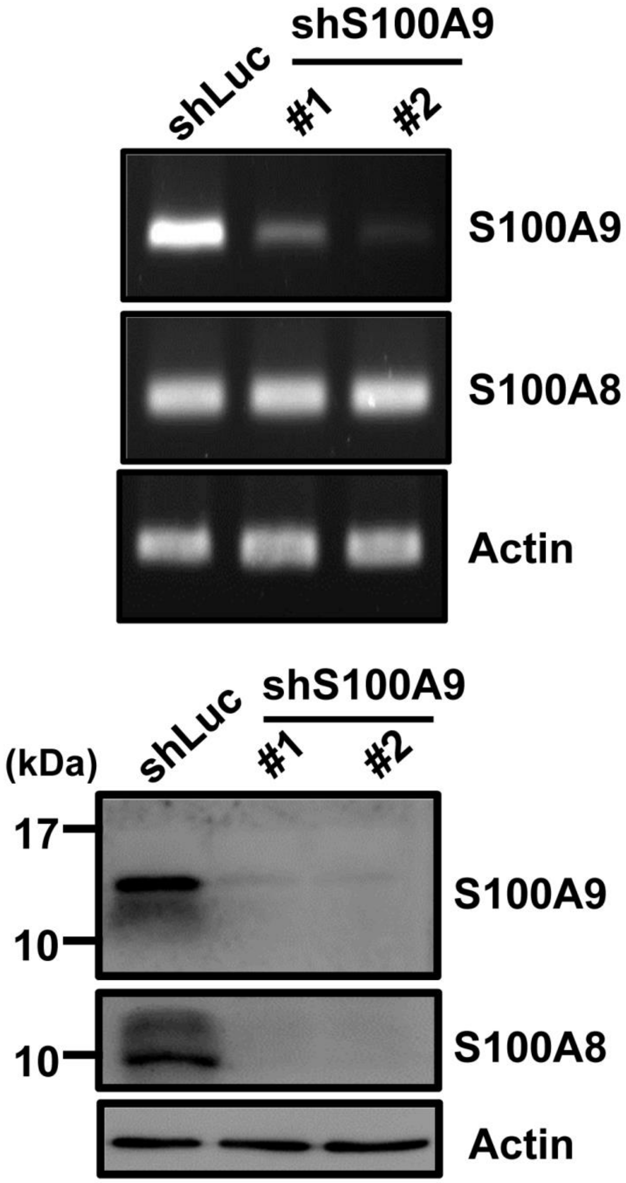

Figure S9 S100A9 knockdown also compromised the expression of S100A8 protein but not mRNA. Oral cancer OC-3 cells were infected for 3 days with S100A9-specific shRNA bearing lentiviruses (clones #1 and #2). Top, the expression of S100A9 but not S100A8 mRNA was specifically reduced in the cells infected with two different clones of shS100A9 measured by semi-quantitative RT-PCR. Bottom, S100A8 protein was also reduced by S100A9-depleted oral cancer cells measured by Western Blot analysis. Actin serves as a loading control.

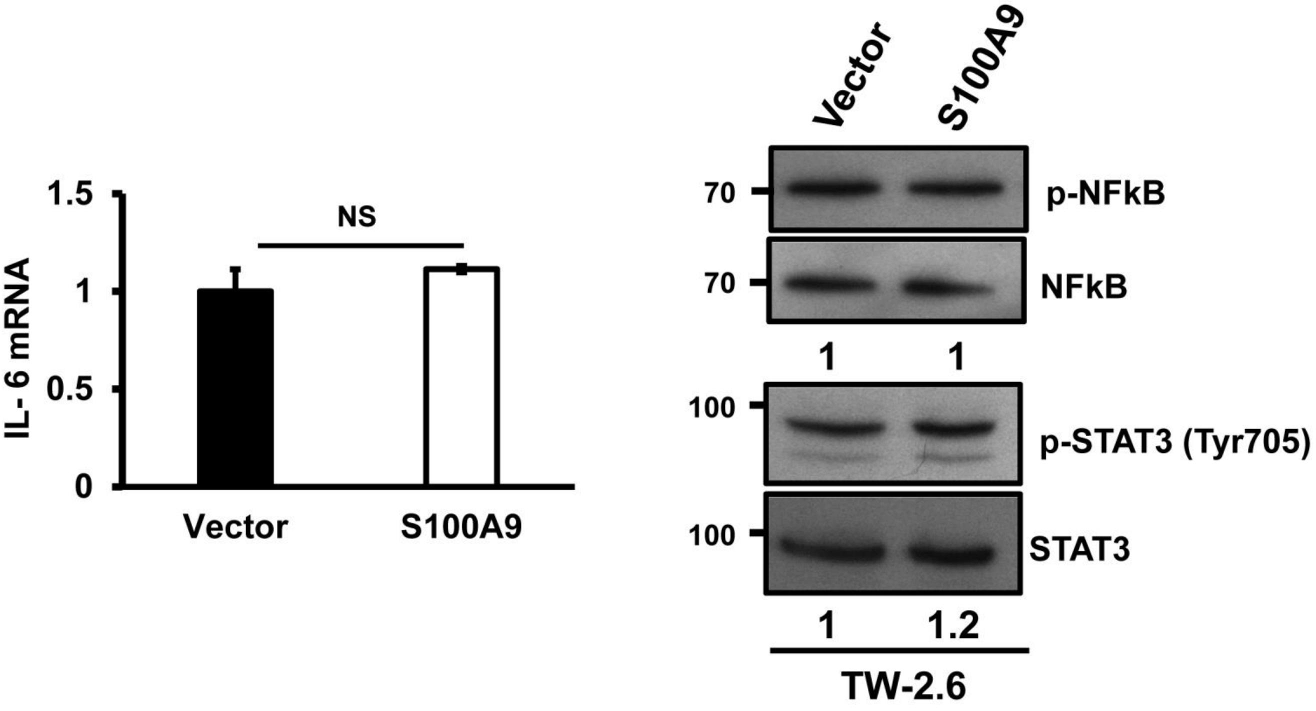

Figure S10 Ectopic S100A9 expression had no effect on IL-6 mRNA expression and its downstream mediators in TW-2.6 cells. Total RNA or protein lysates were isolated from vector or S100A9-expressing TW-2.6 cells. Left, the expression of IL-6 mRNA was measured by qRT-PCR. NS, not significant versus vector. Right, the activating phosphorylation of STAT-3 at Tyr 705 and that of NF-κB in the indicated TW-2.6 cells were analyzed by Western Blot analysis. The numbers underneath are the induction folds.

Table S1 List of primers and their sequence for qRT-PCR analysis

Gene	Primer sequence	
	Human	Murine
IL-6	[F]：5'-GGTACATCCTCGACGGCATCT-3'<br>[R]：5'-GTGCCTCTTTGCTGCTTTCAC-3'	[F]：5'-GACAAAGCCAGAGTCCTTCAG-3'<br>[R]：5'-GTCTTGGTCCTTAGCCACTC-3'
IL-1B	[F]：5'-CCTGTTGTCTACACCAATGC-3'<br>[R]：5'-GGTTGCTCATCAGAATGTGG-3'	[F]：5'-GTCGCTCAGGGTCACAAGAA-3'<br>[R]：5'-GTGCTGCCTAATGTCCCCTT-3'
TNF-α	[F]：5'-CCCAGGGACCTCTCTCTAATC-3'<br>[R]：5'-ATGGGCTACAGGCTTGTCACT-3	[F]：5'-GCCTCTTCTCATTCCTGCTTG-3'<br>[R]：5'-GATGATCTGAGTGTGAGGGTCT-3
THBS1	[F]：5'-ATAAGCTGCTCTGCCCCTTG-3'<br>[R]：5'-AGACCTCATGGCTTTCTTTGC-3'	[F]：5'-TGCTTTTCATCTGGGGCTCA-3'<br>[R]：5'-CCGCCACCTAATTCCACTTG-3'
VEGF-A	[F]：5'-CCTGGTGGACATCTTCCAGGAGTACC-3'<br>[R]：5'-GAAGCTCATCTCTCCTATGTGCTGGC-3'	[F]：5'-GCAGATGTGACAAGCCAAGG-3'<br>[R]：5'-GGTCTTTCCGGTGAGAGGTC-3'
TGFB1	[F]：5'-TGGCGATACCTCAGCAACC-3'<br>[R]：5'-GACAGCTGCTCCACCTTGG-3'	[F]：5'-ACCGCAACAACGCCATCTAT-3'<br>[R]：5'-GCACTGCTTCCCGAATGTCT-3'
IFN-γ	[F]：5'-CTAATTATTCGGTAACTGACTTGA-3'<br>[R]：5'-ACAGTTCAGCCATCACTTGGA-3'	[F]：5'-GCCACGGCACAGTCATTGA-3'<br>[R]：5'-TGCTGATGGCCTGATTGTCTT-3'
MCP-1	[F]：5'-GCCTCCAGCATGAAAGTCTC-3'<br>[R]：5'-AGGTGACTGGGGCATTGAT-3'	[F]：5'-AACTCTCACTGAAGCCAGCTCT-3'<br>[R]：5'-CGTTAACTGCATCTGGCTGA-3'
S100A9	[F]：5'-ACTCTGTGTGGCTCCTCG-3'<br>[R]：5'-TGGTCTCTATGTTGCGTTCC-3'	
CD79a		[F]：5'-CCTGCCTCTCCTCCTCTTCT-3'<br>[R]：5'-AAGTTCACCGTCAGGGATGG-3'
MPO		[F]：5'-ACCCTCATCCAACCCTTCAT-3'<br>[R]：5'-GGTCAATGCCACCTTCCAAC-3'
NK1.1		[F]：5'-TGGACACAGCAAGTATCTACCTCG-3'<br>[R]：5'-GACTCGCACTAAGACACTCATCCC-3'

F4/80		[F]：5’-AGCATCCGAGACACACACAG-3’<br>[R]：5’-GGCAAGACATACCAGGGAGA-3’
Ly6C		[F]：5’-TTCTTCTTGTGGCCCTACTG-3’<br>[R]：5’-AATTGGCACTCCATAGCACTC-3’
Ly6G		[F]：5’-TGCGTTGCTCTGGAGATAGA-3’<br>[R]：5’-TCACGTTGACAGCATTACCA-3’
CD11b		[F]：5’-TTGGCTCTCATCACTGCTGG-3’<br>[R]：5’-TAGACCTGCTTGTTGCTGGG-3’
CD11c		[F]：5’-TGGCTTGTGGTCCTACTGTG-3’<br>[R]：5’-GATGTCTTGGTCTTGCTTTGG-3’
GAPDH	[F]：5’-CCCACTCCTCCACCTTTGA-3’<br>[R]：5’-CCACCACCCTGTTGCTGTAG-3’	[F]：5’-TGTCAAGCTCATTTCCTGGT-3’<br>[R]：5’-TAGGGCCTCTCTTGCTCAGT-3’
